# Development and validation of a scale of healthy psychological effects of physical exercise among Chinese college students from a multidimensional mental health perspective

**DOI:** 10.3389/fpubh.2026.1866486

**Published:** 2026-06-26

**Authors:** Feiyang Liu, Zhengguang Zhu, Rongqiao He, Yu Zhu, Liya Guo

**Affiliations:** 1 School of Physical Education, Southwest University, Chongqing, China; 2 Research Center of Mental Health Education, Southwest University, Chongqing, China

**Keywords:** college students, healthy psychological effects, mental health, physical exercise, scale development

## Abstract

**Background:**

Physical exercise promotes mental health among college students, but existing studies often assess general mental health or limited psychological indicators, making it difficult to capture comprehensive healthy psychological effects after exercise. This study aimed to develop and validate the *Healthy Psychological Effects of Physical Exercise Scale for College Students*.

**Methods:**

An initial scale was developed through theoretical analysis, literature review, semi-structured interviews, and open-ended questionnaires. In an initial sample of 612 college students, item analysis and exploratory factor analysis with varimax rotation were conducted to screen items and identify factor structures. A formal sample of 945 participants was used to examine reliability and validity through Cronbach's α, composite reliability (CR), average variance extracted (AVE), confirmatory factor analysis (CFA), and criterion-related validity testing. Test–retest reliability was assessed in 597 participants after 1 month.

**Results:**

The final scale contained 55 items across five subscales: emotional feelings, self-cognition, frustration coping, self-control and social adaptation efficacy, and interpersonal interaction, comprising 15 subdimensions. Exploratory factor analyses supported clear three-factor structures for each subscale, with cumulative variance explained ranging from 62.546 to 73.711%. CFA indicated good fit for the five subscales (χ^2^*/*df = 2.978–4.391; RMSEA = 0.046–0.060; SRMR = 0.016–0.021; CFI = 0.978–0.988; TLI = 0.971–0.984), and acceptable fit for the overall model *(*χ^2^*/*df = 3.037; RMSEA = 0.046; SRMR = 0.041; CFI = 0.925; TLI = 0.921). Cronbach's α and CR for the 15 subdimensions ranged from 0.873 to 0.931, and AVE ranged from 0.680 to 0.736. Cronbach's α for the five subscale total scores ranged from 0.861 to 0.894. Test–retest reliability ranged from 0.706 to 0.781 for subdimensions and from 0.686 to 0.771 for subscale total scores. Criterion-related validity was supported by significant positive correlations with positive psychological indicators (*r* = 0.229–0.383) and negative correlations with negative psychological indicators (*r* = −0.398 to −0.224; all *p* < 0.001).

**Conclusion:**

The scale demonstrates sound reliability and validity and can be used to assess exercise-related healthy psychological effects among college students.

## 1 Introduction

As emphasized in the definition of health by the World Health Organization, health is not only the absence of disease in the body, but also encompasses mental health, social adaptation, and other multidimensional aspects of overall wellbeing (1). Mental health, as an essential component of health, is closely related to social progress, family harmony, and individual development. It has prominent multidimensional characteristics and has garnered widespread attention from researchers (2). In the context of higher education, physical exercise, as an important lifestyle and effective means of promoting the physical and mental health of college students, plays a crucial role in the multidimensional promotion of mental health. As such, its impact on mental health has become a focal point in health promotion research (3). How to scientifically grasp and effectively measure the psychological changes in college students after physical exercise has thus become an important and ongoing topic of interest for researchers. However, considering that single indicators are relatively straightforward in terms of quantification and can provide clearer research results, and also constrained by the limitations of existing measurement tools that fail to comprehensively reflect the multidimensional aspects of mental health, current studies on the relationship between physical exercise and mental health in college students mainly focus on the changes in single or individual mental health indicators. These studies have yet to systematically present the comprehensive psychological effects that physical exercise may bring. Based on the multidimensional nature of mental health, improving the mental health issues of college students requires attention to the enhancement of overall psychological health, as focusing solely on the improvement of a single mental health indicator has limited practical significance (4). This means that when examining the psychological outcomes of physical exercise in college students, if we remain confined to single indicators, it may be difficult to capture the overall impact of physical exercise on students' mental health, making it harder to provide more explanatory evidence for campus mental health promotion and sports interventions. Therefore, it is necessary to develop a measurement tool from a multidimensional mental health perspective that can systematically reflect the comprehensive psychological effects of physical exercise on college students. This would better reflect the overall mental health status of college students and be more aligned with their psychological health in real-world contexts. Moreover, measuring the psychological changes after physical exercise as a comprehensive psychological effect rather than a change in a single indicator also has clear theoretical and methodological necessity. Firstly, from the structural characteristics of mental health, it is not a simple sum of several individual indicators but the result of the combined effects of positive functional growth and the regulation of negative states (5), with both potentially changing at different magnitudes after physical exercise (6, 7). Secondly, regarding the mechanisms linking physical exercise to college students' mental health, exercise may influence psychological states through multiple pathways, including enhancing positive emotions (8), reducing negative emotions (9), increasing self-control (10), and fostering social connection (11). These multifaceted mechanisms require a measurement framework that matches multidimensional outcomes. Finally, if only a single indicator is used to replace the overall psychological result, it may lead to indicator substitution bias (12), misjudging changes in one dimension as overall psychological benefits, thereby weakening the explanatory power and comparability of the research conclusions.

To ensure clear conceptual boundaries and a consistent measurement orientation in the subsequent scale development, it is necessary to first define “psychological effects” and to introduce the “healthy” orientation adopted in the present study. As research on mental health has continued to deepen, scholars have gradually begun to pay attention to the psychological changes that individuals experience under the influence of specific activities or external conditions. In related studies, the term most commonly used is “psychological benefits,” which refers to the internal experiences or psychological activities obtained by individuals when engaging in a given activity and that are conducive to the development of mental health (13). This conceptual lineage provides important inspiration for the subsequent discussion in the present study. At the same time, from a more general psychological perspective, the cognitive and affective responses generated by individuals in response to external stimuli under specific conditions can also be summarized as “psychological effects” (14). Building on the above definitions, psychological responses in the context of physical exercise likewise possess a neutral attribute. When psychological effects are considered from a health-promotion perspective, “healthy psychological effects” can be understood as psychological changes that are generally conducive to mental health, including the enhancement of positive psychological experiences and adaptive psychological functioning, as well as the alleviation of negative psychological states. However, the concept of “healthy psychological effects of physical exercise” has not yet been clearly and consistently defined in the existing literature. In previous research, when developing the *Adolescent Students' Psychological Benefits of Physical Exercise Scale* as a comprehensive assessment tool, some scholars defined the closely related concept of “psychological benefits of physical exercise” as mainly referring to the psychological gains obtained after exercise (13). The present study is consistent with this orientation; however, in terms of content coverage, it places greater emphasis on the concept as a comprehensive psychological outcome from a “healthy” perspective. Specifically, it includes not only the growth of positive experiences and positive qualities, but also the overall optimization of psychological functioning brought about by the alleviation of negative psychological states. On this basis, drawing on the preceding conceptual definitions, the existing research context, and the specific focus of the present study, “healthy psychological effects of physical exercise” are defined as a type of comprehensive psychological experience and adaptive psychological functioning change that occurs after physical exercise and is directed toward the improvement of mental health. Its core manifestations include the enhancement of positive psychological experiences and adaptive psychological functioning, accompanied by the alleviation of negative psychological states, thereby leading to the overall optimization of psychological wellbeing. This effect is characterized by situational induction and multidimensional integration, and can be structurally measured through self-report items reflecting college students' subjective experiences after physical exercise. Accordingly, the present study regards the healthy psychological effects of physical exercise as an overall variable encompassing multiple dimensions of mental health, and develops a scientific measurement instrument suitable for college students, thereby providing reliable quantitative evidence for related empirical research and practical evaluation.

It should be noted that existing research has also made attempts to examine psychological changes after physical exercise through comprehensive outcomes. However, due to the lack of specialized measurement tools targeting college students that can simultaneously cover both “promotion of positive psychological health” and “alleviation of negative psychological health,” such comprehensive assessments often exhibit clear structural bias in their measurement choices. First, many studies primarily use comprehensive negative psychological symptom indicators to reflect overall mental health levels. In China, early researchers applied standardized symptom tools, such as the “Psychological Health Diagnostic Test,” to explore the relationship between physical exercise and mental health in primary school students (15). This measurement approach for negative psychological symptoms was later extended to the college student population, with standardized symptom tools like SCL-90 being used (16). However, this reveals a significant issue: over-reliance on negative symptoms as proxy indicators for “overall mental health” makes it difficult for the “health promotion” aspects—such as the growth of positive experiences, the development of positive qualities, and the optimization of psychological functions—that physical exercise may bring, to be adequately represented at the measurement level. Second, another line of research has begun to shift toward comprehensive positive psychological indicators (17), emphasizing the role of physical exercise in promoting positive psychological qualities from the perspective of positive psychology. Using positive psychological trait instruments matched to specific educational stages, these studies examine the relationship between physical exercise and positive psychological qualities (18). In addition, some studies conducted in China have taken the concept of “psychological benefits of physical exercise,” which is closely related to the concept adopted in the present study, as the core focus for scale development (13, 19). Nevertheless, such scales have mainly been developed for primary and secondary school students or adolescents, with limited alignment with college students' developmental characteristics and exercise-related psychological experiences. In addition, some studies have employed instruments directly applicable to the college student population, such as the positive mental characters scale for Chinese college students, to explore the relationship between physical exercise and positive psychological qualities among college students (20). Other studies have attempted to use tools that simultaneously include both positive and negative mood dimensions [e.g., the Profile of Mood States (POMS)] to capture bidirectional changes in college students' psychological states following exercise interventions (21). However, such measurement instruments have difficulty comprehensively reflecting the overall psychological changes individuals experience after engaging in physical exercise as a specific activity state. If the psychological effects following physical exercise are not measured as a comprehensive construct, research findings are prone to structural bias. Specifically, when research adopts negative symptoms as the sole measurement orientation, it may underestimate the growth of positive experiences and the improvement of psychological functioning brought about by physical exercise. Conversely, when studies rely exclusively on a single positive variable as the evaluative basis, they may overlook the alleviation of negative psychological states, which represents an equally important dimension of health promotion (22). Similar patterns can also be observed in the evaluation of physical exercise interventions: some programs are more effective in reducing negative emotions but relatively limited in enhancing positive psychological functioning, whereas others show the opposite pattern (23). Furthermore, in the long term, the absence of measurement tools that are directly tailored to college students and that take comprehensive healthy psychological outcomes as their core target will lead different studies to continue using their own single indicators as measurement bases. This results in inconsistent measurement standards, thereby exacerbating the fragmentation of research conclusions, hindering the formation of generalizable evidence chains, and posing persistent challenges to cross-study comparisons and cumulative evidence building.

This further clarifies the key issue that the present study seeks to address: although existing research has accumulated a certain body of evidence along two separate pathways—“symptom alleviation” and “positive enhancement”—there remains a lack, at the measurement level, of a comprehensive instrument suitable for college students that can simultaneously cover both positive enhancement and negative alleviation while also being feasible for large-scale administration. With regard to existing measurement tools, first, instruments commonly used in traditional exercise–mental health research tend to focus on negative symptoms such as anxiety and depression, and pay insufficient attention to the positive aspects of psychological change and the cultivation of positive qualities following physical exercise as a specific activity state. As a result, their scope of consideration is relatively limited, making it difficult to align with the dual characteristics of mental health improvement among college students—namely, the simultaneous reduction of negative psychological experiences and enhancement of positive psychological states—and even more difficult to address both positive enhancement and negative alleviation within a single measurement framework. Second, based on differing conceptual understandings and with reference to comprehensive mental health assessment tools, early researchers preliminarily developed several questionnaires assessing the psychological benefits of physical exercise, thereby advancing related measurement from nonexistence to initial availability. However, most of these instruments were designed for younger adolescent populations. Given the pronounced differences between college students and adolescents at other educational stages in terms of developmental phase, structure of life stressors, and psychological tasks within the Chinese educational context (24), the measurement content and contextual framing of instruments developed for other stages do not necessarily match the college student population. Therefore, the development of a scientifically sound and practically feasible measurement tool targeting college students and assessing the healthy psychological effects of physical exercise is particularly important. Based on the above review, from the perspective of the measurement purpose of the present study, the scope and applicability of existing instruments can be more clearly understood by distinguishing their measurement orientations and target populations. Symptom-oriented tools used in exercise–mental health research, such as the Psychological Health Diagnostic Test and SCL-90, are valuable for assessing negative psychological symptoms; however, their primary purpose is not to comprehensively capture positive psychological enhancement and adaptive functioning after physical exercise (15, 16). Scales of the psychological benefits of physical exercise developed for middle-school students or adolescents provide valuable references for measuring exercise-related psychological outcomes, but their target populations, item contexts, and developmental emphases are not fully aligned with the developmental tasks and exercise-related experiences of college students (13, 19, 24). Instruments focusing on positive psychological qualities among college students are useful for assessing positive psychological resources, but their items are generally not anchored to post-exercise psychological changes (20). Mood-state measures used in exercise intervention studies, such as POMS, can capture affective changes after exercise, but their dimensional coverage is mainly affective and does not sufficiently represent broader domains such as self-cognition, frustration coping, self-control, campus adaptation, and interpersonal functioning within an integrated framework (21). Therefore, although these instruments are valuable for their own measurement purposes, no single existing instrument fully matches the present study's aim of assessing the comprehensive healthy psychological effects of physical exercise among college students. Thus, there is currently no scale specifically targeting the healthy psychological effects of physical exercise among college students, and consequently, there is a lack of empirical research that can systematically reveal the overall healthy psychological effects of physical exercise in this population. In summary, based on a multidimensional perspective and existing research, the present study develops the *Healthy Psychological Effects of Physical Exercise Scale for College Students*, a comprehensive, structurally clear, and administratively feasible instrument specifically designed for the college student population. This scale provides a scientifically sound and effective measurement tool for evaluating the comprehensive healthy psychological effects of physical exercise among college students. At the same time, the scale developed in this study may also serve as a reference or be adapted by scholars in other countries or regions when conducting similar research, offering a reliable instrument for future cross-cultural comparative studies and further promoting international research and practical applications in the field of physical exercise and mental health.

## 2 Preliminary concept of the healthy psychological effects of physical exercise for college students and the construction of the initial scale

### 2.1 Research subjects for the initial scale construction

Both the open-ended questionnaire and semi-structured interviews were conducted with undergraduate students from domestic universities, with a convenience sampling method used to gather the samples. All open-ended questionnaires, semi-structured interviews, and subsequent scale surveys were conducted in Chinese, which was the native language of the participants. The open-ended questionnaire included 90 college students as participants, with 79 valid responses returned, yielding a response rate of 87.78%. Among the valid respondents, 35 were male and 44 were female; 46 were from the humanities and 33 from the sciences. A total of 15 participants were selected for the semi-structured interviews, including nine males and six females; five were from the humanities and 10 from the sciences.

### 2.2 Theoretical basis and development process of the initial scale

Before generating the initial item pool, the present study established a preliminary dimensional framework based on the multidimensional structure of mental health, the dual-factor perspective of mental health, previous research on the psychological benefits of physical exercise, and the developmental characteristics of Chinese college students. On this theoretical and literature-informed basis, the healthy psychological effects of physical exercise among college students were conceptualized as comprehensive post-exercise psychological changes involving emotional experience, self-related cognition, stress and frustration coping, self-regulation and social adaptation, and interpersonal functioning. These five domains reflect the major psychological pathways through which physical exercise may promote mental health and also correspond to the developmental tasks of Chinese college students, including emotion regulation, self-development, academic and life-stress management, behavioral self-control, campus adaptation, and social connection.

Accordingly, five preliminary subscales—emotional feelings, self-cognition, frustration coping, self-control and social adaptation efficacy, and interpersonal interaction—were specified *a priori*. These subscales were intended to represent five interrelated domains of exercise-related healthy psychological changes: affective experience, self-evaluation, coping-related psychological functioning, self-regulation and adaptation efficacy, and interpersonal functioning. Within this framework, the fifteen subdimensions were further organized as more specific manifestations under the five theoretically proposed domains. Their operational definitions are presented in Tables 1 and 2.

**Table 1:** Subscale composition and definitions.

Subscale	Variable definition
Emotional feelings	The emotional experiences of college students after physical exercise that benefit mental health, including the enhancement of positive emotions, increase in restorative calmness, and alleviation of negative emotional distress.
Self-cognition	The subjective changes in self-assessment related to personal abilities, value and strengths, as well as perceptions of body function and appearance after physical exercise.
Frustration coping	The subjective coping ability and psychological adaptation level of college students in dealing with stress and setbacks, including attention control, emotional regulation, cognitive flexibility, and positive expectation after physical exercise.
Self-control and social adaptation efficacy	The changes in self-regulation and adaptation efficacy in key scenarios such as academic task management, self-control of electronic entertainment behavior, and integration into campus life following physical exercise.
Interpersonal interaction	The subjective improvements in interpersonal communication competence, relationship security, support experience, and proactive social interaction and prosocial tendencies after physical exercise.

**Table 2:** Subdimension composition and definitions.

Subscale	Subdimension	Variable definition
Emotional feelings	Psychological positive motivation	The increased enthusiasm, vitality, and enjoyment experienced by college students after physical exercise, manifested as enhanced positive emotions and motivation.
	Psychological calmness	The inner ease, calmness, and harmony experienced by college students after physical exercise, along with emotional stability and relaxation.
	Emotional disturbance	The reduction in negative emotions and psychological distress experienced by college students after physical exercise.
Self-cognition	Competence efficacy	The positive judgment and confident experience of college students regarding their own abilities and potential for success after physical exercise.
	Self-acceptance	The positive affirmation of personal value, strengths, and advantages, as well as the tendency to maintain self-position in social comparison or evaluation situations after physical exercise.
	Body self-perception	The subjective improvement in the vitality of body functions and satisfaction with physical appearance and attractiveness after physical exercise.
Frustration coping	Concentration of energy	The psychological adaptability of college students to regulate emotions, eliminate distractions, and focus on tasks and problem-solving effectively when facing setbacks.
	Flexible thinking	The ability of college students to adjust their thinking styles, change perspectives, and maintain a calm mindset to cope effectively with difficulties after physical exercise.
	Optimistic mindset	The tendency of college students to maintain a positive attitude toward challenges, believing in their ability to find solutions and improve the situation after physical exercise.
Self-control and social adaptation efficacy	Academic coping	The positive changes observed in college students, such as improved attitude, increased concentration, and enhanced time management efficiency when completing academic tasks after physical exercise.
	Addiction control	The ability of college students to manage and control their dependency and impulsivity toward activities such as the internet, social media, or video games after physical exercise.
	Campus adaptation	The ability of college students to integrate better into campus life by effectively utilizing resources, balancing academic and personal life, and actively participating in campus activities after physical exercise.
Interpersonal interaction	Interpersonal communication	The subjective improvements in communication skills, adaptability, patience, and conflict resolution abilities when interacting with others after physical exercise.
	Relationship perception	The cognitive changes and psychological experiences related to the perception of relationships with others during interactions after physical exercise.
	Willingness to interact	The internal motivation and positive attitude of college students to actively communicate, establish connections, offer help, and display prosocial behaviors after physical exercise.

Subsequently, semi-structured interviews and open-ended questionnaires were used to supplement, refine, and contextualize the preliminary framework at the empirical level. The semi-structured interviews collected college students' in-depth descriptions of their post-exercise psychological experiences, while the open-ended questionnaire further examined the coverage of the proposed dimensions and the comprehensibility of item expressions. Students with long-term physical exercise experience were purposively invited to obtain richer and more concrete descriptions of exercise-related psychological changes. However, the interview materials were used mainly to refine the theory- and literature-informed framework and item wording, rather than to independently determine the scale structure. The applicability of the preliminary structure to ordinary college students was subsequently examined through the open-ended questionnaire and quantitative validation in broader college student samples. Expert consultation was then conducted with scholars in psychology, education, and related fields, as well as experienced university physical education instructors, to evaluate the conceptual relevance, structural rationality, and wording appropriateness of the subscales, subdimensions, and items.

Finally, based on the theoretical framework, qualitative materials, open-ended questionnaire results, and expert consultation, the initial item pool was revised and optimized. Faculty members and doctoral students in sport psychology and health psychology further evaluated the content representativeness of the items, while doctoral students majoring in Chinese language and literature assessed the accuracy and semantic clarity of the item wording. A small-scale pilot test was also conducted to identify items with insufficient representativeness, confusing wording, or ambiguous meanings. Through this sequential process of theoretical construction, qualitative refinement, expert evaluation, and item optimization, the initial version of the *Healthy Psychological Effects of Physical Exercise Scale for College Students* was formed.

### 2.3 Results of the initial scale construction

Following the above process of preliminary framework development, qualitative refinement, expert consultation, and item optimization, the initial version of the *Healthy Psychological Effects of Physical Exercise Scale for College Students* was determined to consist of five subscales: emotional feelings, self-cognition, frustration coping, self-control and social adaptation efficacy, and interpersonal interaction. Based on the measurement objectives of each subscale, these were further refined into 15 subdimensions: psychological positive motivation, psychological calmness, emotional disturbance, competence efficacy, self-acceptance, body self-perception, concentration of energy, flexible thinking, optimistic mindset, academic coping, addiction control, campus adaptation, interpersonal communication, relationship perception, and willingness to interact. The specific breakdown is shown in Tables 1 and 2.

After the preliminary conceptualization of the *Healthy Psychological Effects of Physical Exercise Scale for College Students*, consisting of five subscales and 15 subdimensions, and based on the literature review, semi-structured interviews, and open-ended survey results, this study developed the initial scale for measuring the healthy psychological effects of physical exercise in college students. The scale comprises 75 items, distributed as follows:

- Emotional feelings subscale (12 items):
  • Psychological positive motivation subdimension (three items)
  • Psychological calmness subdimension (three items)
  • Emotional disturbance subdimension (six items)

- Self-cognition subscale (14 items):
  • Competence efficacy subdimension (five items)
  • Self-acceptance subdimension (six items)
  • Body self-perception subdimension (three items)

- Frustration coping subscale (15 items):
  • Concentration of energy subdimension (five items)
  • Flexible thinking subdimension (four items)
  • Optimistic mindset subdimension (six items)

- Self-control and social adaptation efficacy subscale (18 items):
  • Academic coping subdimension (six items)
  • Addiction control subdimension (six items)
  • Campus adaptation subdimension (six items)

- Interpersonal interaction subscale (16 items):
  • Interpersonal communication subdimension (five items)
  • Relationship perception subdimension (four items)
  • Willingness to interact subdimension (seven items)

Following the scoring method established by scholars such as Zhou Chenglin for the *Adolescent Students' Psychological Benefits of Physical Exercise Scale*, this study's scale uses a Likert five-point scoring system (13). The scoring system is as follows:

- Five points: completely agree
- Four points: agree
- Three points: uncertain
- Two points: disagree
- One point: completely disagree

All items are single-choice questions.

## 3 Initial testing of the healthy psychological effects of physical exercise scale for college students

By administering the scale and conducting statistical analyses, this study explored and verified the empirical structure and the theoretical structure of the healthy psychological effects of physical exercise among college students. On this basis, the formal version of the *Healthy Psychological Effects of Physical Exercise Scale for College Students* was developed, providing a scientifically sound and reasonable measurement tool for assessing the healthy psychological effects of physical exercise in this population.

### 3.1 Participants in the initial testing

Following the principle of stratified cluster sampling, this study selected a total of three institutions from the eastern, central, and western regions of China to conduct the survey, yielding 612 valid responses. The sample covered multiple demographic variables. In terms of gender distribution, there were 295 males (48.2%) and 317 females (51.8%). The participants had a mean age of 19.81 years (SD = 1.40), indicating that most participants' ages were relatively concentrated. Regarding place of family residence, participants came from diverse areas, including provincial capitals (*n* = 154, 25.2%), prefecture-level cities or autonomous prefectures (*n* = 127, 20.8%), county-level cities/towns (*n* = 155, 25.3%), townships (*n* = 65, 10.6%), and rural areas (*n* = 111, 18.1%). Concerning only-child status, 261 participants (42.6%) were only children, whereas 351 (57.4%) were not. Grade distribution showed that 177 students were freshmen (28.9%), 165 were sophomores (27.0%), 136 were juniors (22.2%), and 134 were seniors (21.9%). Overall, the sample demonstrated a relatively broad distribution in gender, age, family background, and grade level, which could adequately reflect the basic demographic characteristics of contemporary college students and thus possesses a certain degree of representativeness.

### 3.2 Analysis strategy and statistical tools for the initial scale testing

In this study, multiple statistical methods were employed to ensure the reliability, validity, and item quality of the scale, including descriptive statistics, reliability analysis, item analysis, and exploratory factor analysis (EFA). First, descriptive statistics were used to summarize the demographic characteristics of the sample and the basic features of the scale variables. This approach helped to describe fundamental information such as gender and age distributions and to compute descriptive indices of the scale scores, including means and standard deviations. Second, item analysis was conducted to evaluate the quality of each item in the scale. Specifically, item performance was assessed using the critical ratio, item–total correlation (ITC), and changes in Cronbach's α if an item was deleted. Items with low critical ratio values, insufficient item–total correlations, or a substantial increase in Cronbach's α upon deletion were considered for removal. This procedure ensured that each item made an adequate contribution to the scale and enhanced its overall reliability. Through these indices, the reliability of the scale was systematically evaluated. EFA was employed to identify the underlying factor structure of the scale. Through EFA, the factor loadings of items on different factors were examined to determine item quality and retention decisions. The results of the EFA provided a preliminary structural basis for subsequent confirmatory analyses. Throughout the study, SPSS 29.0 (IBM Corp., Armonk, NY, USA) was used to perform descriptive statistics, item analysis, reliability analysis, and EFA.

### 3.3 Results of the initial scale testing

#### 3.3.1 Item analysis of the initial scale testing

To ensure the reliability and validity of the scale, item quality was evaluated based on critical ratios, item–total correlations, and changes in Cronbach's α after item deletion. Specifically, if the critical ratios of all items reached a significant level (*p* < 0.001), this would indicate that the items had good discriminative power between the high- and low-score groups. If the item–total correlation coefficients were all greater than 0.40, this would suggest that the items were significantly correlated with the total scale score. Furthermore, changes in Cronbach's α after item deletion were examined to determine whether removing specific items would improve the overall reliability of the scale.

## 3.3.1.1 Item analysis results of the emotional feelings subscale

As shown in Table 3, none of the three subdimensions within the emotional feelings subscale exhibited items of insufficient quality. Specifically, the critical ratios of all items reached statistically significant levels, and all items showed significant positive correlations with the total scores of their respective subdimensions. Moreover, the deletion of any item did not result in a noticeable decrease in Cronbach's α, indicating that all items contributed adequately to the internal consistency of the subscale.

**Table 3:** Critical ratios and item–total correlations of items for each subdimension of the emotional feelings subscale.

Subdimension	Item no.	Critical ratio	Item–total correlation	Cronbach's α if item deleted	Cronbach's α
Psychological positive motivation	Emotional feelings 1	33.355^***^	0.709^***^	0.747	0.831
	Emotional feelings 3	30.664^***^	0.677^***^	0.778	
	Emotional feelings 4	35.076^***^	0.683^***^	0.772	
Psychological calmness	Emotional feelings 9	37.220^***^	0.710^***^	0.783	0.844
	Emotional feelings 10	36.478^***^	0.738^***^	0.755	
	Emotional feelings 12	30.619^***^	0.683^***^	0.808	
Emotional disturbance	Emotional feelings 2	32.428^***^	0.745^***^	0.888	0.906
	Emotional feelings 5	33.209^***^	0.755^***^	0.887	
	Emotional feelings 6	28.361^***^	0.731^***^	0.890	
	Emotional feelings 7	34.793^***^	0.737^***^	0.890	
	Emotional feelings 8	32.777^***^	0.754^***^	0.887	
	Emotional feelings 11	29.171^***^	0.720^***^	0.892	

^***^p < 0.001.

## 3.3.1.2 Item analysis results of the self-cognition subscale

As shown in Table 4, two subdimensions within the self-cognition subscale contained items of relatively low quality. Specifically, in the competence efficacy subdimension, Cronbach's α increased significantly to 0.877 after deleting item self-cognition 3. In the Self-Acceptance subdimension, the deletion of items self-cognition 5, 13, and 14 resulted in an increase in Cronbach's α, with the coefficient reaching 0.831 after the removal of these three items. In contrast, all items within the body self-perception subdimension demonstrated satisfactory quality and were therefore retained.

**Table 4:** Critical ratios and item–total correlations of items for each subdimension of the self-cognition subscale.

Subdimension	Item no.	Critical ratio	Item–total correlation	Cronbach's α if item deleted	Cronbach's α
Competence efficacy	Self-cognition 1	32.330^***^	0.710^***^	0.756	0.821
	Self-cognition 3	10.977^***^	0.246^***^	0.877	
	Self-cognition 4	33.641^***^	0.729^***^	0.750	
	Self-cognition 10	32.930^***^	0.713^***^	0.754	
	Self-cognition 11	29.971^***^	0.694^***^	0.760	
Self-acceptance	Self-cognition 2	22.981^***^	0.486^***^	0.293	0.473
	Self-cognition 5	8.691^***^	0.053	0.531	
	Self-cognition 9	21.631^***^	0.476^***^	0.302	
	Self-cognition 12	22.552^***^	0.486^***^	0.300	
	Self-cognition 13	9.765^***^	0.047	0.535	
	Self-cognition 14	9.984^***^	0.055	0.532	
Body self-perception	Self-cognition 6	35.699^***^	0.682^***^	0.736	0.817
	Self-cognition 7	30.637^***^	0.658^***^	0.761	
	Self-cognition 8	32.288^***^	0.669^***^	0.749	

^***^p < 0.001.

## 3.3.1.3 Item analysis results of the frustration coping subscale

As shown in Table 5, within the frustration coping subscale, each of the three subdimensions contained some items of relatively low quality. Specifically, in the concentration of energy subdimension, Cronbach's α increased significantly to 0.871 after deleting item frustration coping 13. In the Flexible Thinking subdimension, Cronbach's α increased significantly to 0.824 after deleting item frustration coping 10. In the optimistic mindset subdimension, Cronbach's α increased significantly to 0.865 after deleting item frustration coping 12.

**Table 5:** Critical ratios and item–total correlations of items for each subdimension of the frustration coping subscale.

Subdimension	Item no.	Critical ratio	Item–total correlation	Cronbach's α if item deleted	Cronbach's α
Concentration of energy	Frustration coping 1	28.030^***^	0.649^***^	0.630	0.734
	Frustration coping 2	28.209^***^	0.652^***^	0.630	
	Frustration coping 3	30.302^***^	0.656^***^	0.627	
	Frustration coping 7	33.642^***^	0.683^***^	0.612	
	Frustration coping 13	8.669^***^	0.028	0.871	
Flexible thinking	Frustration coping 5	29.619^***^	0.660^***^	0.650	0.761
	Frustration coping 6	31.880^***^	0.657^***^	0.649	
	Frustration coping 10	13.843^***^	0.307^***^	0.824	
	Frustration coping 14	30.725^***^	0.640^***^	0.659	
Optimistic mindset	Frustration coping 4	20.375^***^	0.547^***^	0.756	0.789
	Frustration coping 8	28.176^***^	0.656^***^	0.730	
	Frustration coping 9	27.405^***^	0.661^***^	0.728	
	Frustration coping 11	31.752^***^	0.739^***^	0.710	
	Frustration coping 12	10.467^***^	0.132^**^	0.865	
	Frustration coping 15	27.505^***^	0.657^***^	0.730	

^**^p < 0.01, ^***^p < 0.001.

## 3.3.1.4 Item analysis results of the self-control and social adaptation efficacy subscale

As shown in Table 6, all three subdimensions within the self-control and social adaptation efficacy subscale contained some items of relatively low quality. Specifically, in the Academic coping subdimension, Cronbach's α increased significantly to 0.749 after deleting items efficacy 12 and efficacy 13. In the addiction control subdimension, Cronbach's α increased significantly to 0.873 after deleting the corresponding low-quality item(s). In the campus adaptation subdimension, Cronbach's α increased significantly to 0.889 after deleting items efficacy 17 and efficacy 18.

**Table 6:** Critical ratios and item–total correlations of items for each subdimension of the self-control and social adaptation efficacy subscale.

Subdimension	Item no.	Critical ratio	Item–total correlation	Cronbach's α if item deleted	Cronbach's α
Academic coping	Efficacy 1	12.386^***^	0.248^***^	0.529	0.553
	Efficacy 2	25.115^***^	0.548^***^	0.395	
	Efficacy 3	25.067^***^	0.503^***^	0.414	
	Efficacy 10	22.911^***^	0.509^***^	0.412	
	Efficacy 12	11.096^***^	0.116^**^	0.599	
	Efficacy 13	6.853^***^	0.017	0.642	
Addiction control	Efficacy 4	28.460^***^	0.665^***^	0.694	0.768
	Efficacy 5	6.406^***^	−0.005	0.873	
	Efficacy 6	32.464^***^	0.758^***^	0.672	
	Efficacy 7	20.480^***^	0.529^***^	0.730	
	Efficacy 8	27.763^***^	0.669^***^	0.694	
	Efficacy 9	27.315^***^	0.668^***^	0.693	
Campus adaptation	Efficacy 11	28.217^***^	0.628^***^	0.575	0.685
	Efficacy 14	25.635^***^	0.621^***^	0.576	
	Efficacy 15	30.193^***^	0.679^***^	0.555	
	Efficacy 16	29.792^***^	0.636^***^	0.573	
	Efficacy 17	6.932^***^	0.033	0.779	
	Efficacy 18	9.851^***^	0.135^***^	0.739	

^**^p < 0.01, ^***^p < 0.001.

In this table, self-control and social adaptation efficacy is abbreviated as efficacy.

## 3.3.1.5 Item analysis results of the interpersonal interaction subscale

As shown in Table 7, two subdimensions within the interpersonal interaction subscale contained items of relatively low quality, whereas no items of poor quality were identified in the interpersonal communication subdimension. Specifically, in the relationship perception subdimension, Cronbach's α increased significantly to 0.813 after deleting item interpersonal interaction 12. In the willingness to interact subdimension, Cronbach's α increased significantly to 0.810 after deleting item interpersonal interaction 6.

**Table 7:** Critical ratios and item–total correlations of items for each subdimension of the interpersonal interaction subscale.

Subdimension	Item no.	Critical ratio	Item–total correlation	Cronbach's α if item deleted	Cronbach's α
Interpersonal communication	Interpersonal interaction 1	29.544^***^	0.685^***^	0.821	0.855
	Interpersonal interaction 3	28.696^***^	0.683^***^	0.822	
	Interpersonal interaction 5	37.021^***^	0.769^***^	0.798	
	Interpersonal interaction 15	23.061^***^	0.578^***^	0.849	
	Interpersonal interaction 16	26.118^***^	0.636^***^	0.834	
Relationship perception	Interpersonal interaction 9	28.699^***^	0.590^***^	0.378	0.605
	Interpersonal interaction 10	28.775^***^	0.556^***^	0.404	
	Interpersonal interaction 11	25.358^***^	0.522^***^	0.435	
	Interpersonal interaction 12	9.562^***^	0.024	0.813	
Willingness to interact	Interpersonal interaction 2	12.648^***^	0.366^***^	0.736	0.747
	Interpersonal interaction 4	28.487^***^	0.667^***^	0.669	
	Interpersonal interaction 6	7.692^***^	0.086^*^	0.810	
	Interpersonal interaction 7	27.457^***^	0.659^***^	0.673	
	Interpersonal interaction 8	11.210^***^	0.313^***^	0.746	
	Interpersonal interaction 13	27.084^***^	0.636^***^	0.675	
	Interpersonal interaction 14	27.871^***^	0.642^***^	0.674	

^*^p < 0.05, ^***^p < 0.001.

### 3.3.2 Exploratory factor analysis of the initial scale testing

To evaluate the structural validity of the scale and ensure item quality, exploratory factor analysis (EFA) was conducted based on the results of the item analysis. First, Bartlett's test of sphericity and the Kaiser–Meyer–Olkin (KMO) measure were employed to determine whether the data were suitable for factor analysis. Bartlett's test of sphericity was used to examine whether the correlations among variables were sufficiently strong; a significant result indicates that the data are appropriate for factor analysis. Meanwhile, the KMO value was used to assess sampling adequacy, with values greater than 0.60 indicating suitability for EFA. In the present study, Bartlett's test was significant and the KMO value reached an ideal level, suggesting that the data met the prerequisites for factor analysis. During factor extraction, principal component analysis was adopted, and varimax rotation was applied to clarify the underlying factor structure. Although factor loadings above 0.40 are often regarded as acceptable in exploratory factor analysis, the cutoff value should be determined according to the purpose of scale development, sample size, expected factor clarity, and the balance between parsimony and construct coverage. Methodological literature suggests that factor loading cutoffs should be interpreted in light of sample size, factor stability, and the purpose of scale development, and that item retention should also consider theoretical relevance and content representativeness (25, 26). Based on these considerations, the present study adopted 0.60 as the primary loading criterion to improve the parsimony, interpretability, and psychometric quality of the formal scale. Item retention and deletion were determined by jointly considering primary factor loadings, cross-loading patterns, item analysis results, consistency with the theoretical framework, expert evaluation, and content representativeness. Items with relatively low loadings were deleted only when they also showed weak item quality, unclear factor membership, substantial cross-loading, theoretical mismatch, or content redundancy.

#### 3.3.2.1 Exploratory factor analysis results of the emotional feelings subscale

The KMO measure yielded a value of 0.866, indicating that the data were highly suitable for factor extraction. Bartlett's test of sphericity was significant (χ^2^ = 3,703.263, df = 66, *p* < 0.001), further confirming sufficient correlations among variables and the appropriateness of conducting factor analysis. Factor extraction for this subscale identified three common factors, with a cumulative variance explained of 71.830%, exceeding the 60% criterion and indicating that the three factors accounted for the major variance of the items in this subscale. Moreover, the number of extracted factors was consistent with the previously proposed three subdimensions at the theoretical level, providing a basis for subsequent examination of item allocation and structural consistency based on the rotated factor loading matrix. To obtain a clearer factor structure, orthogonal rotation using the varimax method was applied to the factor loading matrix. In selecting measurement items, the magnitude of factor loadings was used as the criterion for item retention or deletion. Items with factor loadings below 0.60 were removed, and items loading on the same column in the final factor solution were classified into the same factor. The results are presented in Table 8.

**Table 8:** Rotated factor loading matrix of the emotional feelings subscale.

Item	Factor loading coefficients		
	Factor 1	Factor 2	Factor 3
Emotional feelings 1	0.139	0.112	0.858
Emotional feelings 3	0.094	0.093	0.850
Emotional feelings 4	0.133	0.134	0.836
Emotional feelings 9	0.117	0.857	0.112
Emotional feelings 10	0.134	0.873	0.112
Emotional feelings 12	0.094	0.844	0.115
Emotional feelings 2	0.826	0.053	0.086
Emotional feelings 5	0.830	0.082	0.078
Emotional feelings 6	0.803	0.088	0.120
Emotional feelings 7	0.804	0.103	0.136
Emotional feelings 8	0.824	0.140	0.059
Emotional feelings 11	0.794	0.093	0.112

According to the rotated factor loading matrix, item allocation was well-concentrated and the factor structure was clear. Specifically, Factor 1 consisted of items emotional feelings 2, 5, 6, 7, 8, and 11, and the content of these items corresponded to the Emotional Disturbance subdimension proposed in the theoretical framework. Factor 2 comprised items emotional feelings 9, 10, and 12, whose content aligned with the Psychological Calmness subdimension proposed earlier. Factor 3 included items emotional feelings 1, 3, and 4, and their content corresponded to the Psychological Positive Motivation subdimension in the theoretical model. All items showed primary factor loadings above 0.60 on their respective factors, with relatively low loadings on other factors, and no notable cross-loadings were observed. Overall, the three-factor structure extracted in this exploratory factor analysis was highly consistent with the proposed theoretical framework. This not only provides exploratory structural validity support for the subdimension classification of this subscale but also empirically confirms the rationality and interpretability of the preliminary theoretical framework. Accordingly, no items were identified for deletion in this round of analysis, and all items were retained for subsequent analyses.

#### 3.3.2.2 Exploratory factor analysis results of the self-cognition subscale

The KMO measure yielded a value of 0.801, and Bartlett's test of sphericity was statistically significant (χ^2^ = 2,661.571, df = 45, *p* < 0.001), indicating that the data were suitable for factor analysis. Factor extraction for this subscale identified three common factors, with a cumulative variance explained of 73.711%. Subsequently, orthogonal rotation using the varimax method was applied to the factor loading matrix. The results are presented in Table 9.

**Table 9:** Rotated factor loading matrix of the self-cognition subscale.

Item	Factor loading coefficients		
	Factor 1	Factor 2	Factor 3
Self-cognition 1	0.853	0.064	0.100
Self-cognition 4	0.853	0.066	0.100
Self-cognition 10	0.840	0.056	0.124
Self-cognition 11	0.836	0.082	0.088
Self-cognition 2	0.126	0.839	0.102
Self-cognition 9	0.025	0.854	0.149
Self-cognition 12	0.070	0.875	0.033
Self-cognition 6	0.111	0.086	0.853
Self-cognition 7	0.134	0.094	0.830
Self-cognition 8	0.095	0.101	0.845

According to the rotated factor loading matrix, item allocation was well-concentrated and the factor structure was clear. Specifically, Factor 1 consisted of items Self-cognition 1, 4, 10, and 11, corresponding to the competence efficacy subdimension proposed in the theoretical framework. Factor 2 comprised items self-cognition 2, 9, and 12, corresponding to the self-acceptance subdimension. Factor 3 included items self-cognition 6, 7, and 8, corresponding to the body self-perception subdimension. All items exhibited primary factor loadings greater than 0.60 on their respective factors, with relatively low loadings on other factors, and no notable cross-loadings were observed. Overall, consistent with the results for the emotional feelings subscale, the three-factor structure extracted in this exploratory factor analysis was highly consistent with the proposed theoretical framework, providing exploratory evidence of structural validity for the subdimension classification of this subscale. Accordingly, no items were identified for deletion in this round of analysis, and all items were retained for subsequent analyses.

#### 3.3.2.3 Exploratory factor analysis results of the frustration coping subscale

The KMO measure yielded a value of 0.813, and Bartlett's test of sphericity was statistically significant (χ^2^ = 3,654.788, df = 66, *p* < 0.001), indicating that the data were suitable for factor analysis. Factor extraction for this subscale identified three common factors, with a cumulative variance explained of 70.568%. Orthogonal rotation using the varimax method was applied to the factor loading matrix, and the results are presented in Table 10.

**Table 10:** Rotated factor loading matrix of the frustration coping subscale.

Item	Factor loading coefficients		
	Factor 1	Factor 2	Factor 3
Frustration coping 1	0.100	0.828	0.116
Frustration coping 2	0.100	0.842	0.032
Frustration coping 3	0.067	0.842	0.083
Frustration coping 7	0.114	0.847	0.102
Frustration coping 5	0.120	0.096	0.838
Frustration coping 6	0.138	0.114	0.853
Frustration coping 14	0.070	0.064	0.836
Frustration coping 4	0.617	0.108	0.442
Frustration coping 8	0.819	0.096	0.056
Frustration coping 9	0.809	0.082	0.079
Frustration coping 11	0.859	0.117	0.087
Frustration coping 15	0.820	0.068	0.083

Based on the factor loading matrix, all items except frustration coping 4 exhibited relatively high primary loadings on their respective factors and low loadings on other factors, with no notable cross-loadings, indicating good item discrimination. Specifically, Factor 2 consisted of items frustration coping 1, 2, 3, and 7, corresponding to the concentration of energy subdimension proposed in the theoretical framework; Factor 3 comprised items frustration coping 5, 6, and 14, corresponding to the flexible thinking subdimension; and Factor 1 mainly included items frustration coping 8, 9, 11, and 15, corresponding to the optimistic mindset subdimension. It should be noted that frustration coping 4 showed relatively high loadings on both Factor 1 and Factor 3, with a difference of less than 0.20, indicating substantial cross-loading and unclear factor membership. This item was therefore deleted to improve the parsimony and interpretability of the factor structure. All remaining items met the retention criteria and were retained for subsequent analyses.

#### 3.3.2.4 Exploratory factor analysis results of the self-control and social adaptation efficacy subscale

The KMO measure yielded a value of 0.835, and Bartlett's test of sphericity was statistically significant (χ^2^ = 4,058.497, df = 78, *p* < 0.001), indicating that the data were suitable for factor analysis. Factor extraction for this subscale identified three common factors, with a cumulative variance explained of 68.139%. Orthogonal rotation using the varimax method was applied to the factor loading matrix, and the results are presented inTable 11.

**Table 11:** Rotated factor loading matrix of the self-control and social adaptation efficacy subscale.

Item	Factor loading coefficients		
	Factor 1	Factor 2	Factor 3
Self-control and social adaptation efficacy 1	0.099	0.115	0.386
Self-control and social adaptation efficacy 2	0.098	0.104	0.854
Self-control and social adaptation efficacy 3	0.191	0.099	0.829
Self-control and social adaptation efficacy 10	0.065	0.090	0.854
Self-control and social adaptation efficacy 4	0.810	0.054	0.180
Self-control and social adaptation efficacy 6	0.890	0.071	0.076
Self-control and social adaptation efficacy 7	0.640	0.450	0.058
Self-control and social adaptation efficacy 8	0.806	0.037	0.154
Self-control and social adaptation efficacy 9	0.822	0.059	0.130
Self-control and social adaptation efficacy 11	0.076	0.847	0.093
Self-control and social adaptation efficacy 14	0.084	0.835	0.139
Self-control and social adaptation efficacy 15	0.108	0.879	0.091
Self-control and social adaptation efficacy 16	0.070	0.831	0.188

According to the rotated factor loading matrix, self-control and social adaptation efficacy 1 showed relatively low loadings across all three factors, with insufficient primary loadings, making it difficult to assign this item to any latent factor. In addition, self-control and social adaptation efficacy 7 exhibited relatively high loadings on both Factor 1 and Factor 2, with a difference of less than 0.20, indicating pronounced cross-loading and unclear factor membership. Therefore, self-control and social adaptation efficacy 1 and self-control and social adaptation efficacy 7 were deleted in this round of analysis. The remaining items demonstrated clear loading patterns. Specifically, Factor 3 consisted of items self-control and social adaptation efficacy 2, 3, and 10, corresponding to the academic coping subdimension proposed in the theoretical framework; Factor 1 included items self-control and social adaptation efficacy 4, 6, 8, and 9, corresponding to the addiction control subdimension; and Factor 2 comprised items self-control and social adaptation efficacy 11, 14, 15, and 16, corresponding to the campus adaptation subdimension. Accordingly, with the exception of the deleted items, all remaining items were retained and entered into subsequent analyses.

#### 3.3.2.5 Exploratory factor analysis results of the interpersonal interaction subscale

The KMO measure yielded a value of 0.850, and Bartlett's test of sphericity was statistically significant (χ^2^ = 3,804.667, df = 91, *p* < 0.001), indicating that the data were suitable for factor analysis. Factor extraction for this subscale identified three common factors, with a cumulative variance explained of 62.546%. Orthogonal rotation using the varimax method was applied to the factor loading matrix, and the results are presented in Table 12.

**Table 12:** Rotated factor loading matrix of the interpersonal interaction subscale.

Item	Factor loading coefficients		
	Factor 1	Factor 2	Factor 3
Interpersonal interaction 1	0.812	0.149	0.062
Interpersonal interaction 3	0.776	0.169	0.155
Interpersonal interaction 5	0.840	0.104	0.141
Interpersonal interaction 15	0.599	0.057	0.513
Interpersonal interaction 16	0.777	0.167	0.039
Interpersonal interaction 9	0.150	0.126	0.855
Interpersonal interaction 10	0.093	0.191	0.791
Interpersonal interaction 11	0.094	0.157	0.808
Interpersonal interaction 2	0.311	0.337	0.295
Interpersonal interaction 4	0.098	0.846	0.065
Interpersonal interaction 7	0.113	0.812	0.121
Interpersonal interaction 8	0.163	0.376	0.205
Interpersonal interaction 13	0.112	0.794	0.171
Interpersonal interaction 14	0.144	0.819	0.083

According to the rotated factor loading matrix, interpersonal interaction 2 and interpersonal interaction 8 showed relatively low loadings across all factors, making it difficult to assign them to any latent factor. In addition, interpersonal interaction 15 exhibited relatively high loadings on both Factor 1 and Factor 3, with a small difference between the two, indicating substantial cross-loading and unclear factor membership. Accordingly, interpersonal interaction 2, 8, and 15 were deleted in this round of analysis. The remaining items demonstrated relatively clear loading patterns. Specifically, Factor 1 mainly consisted of items interpersonal interaction 1, 3, 5, and 16, corresponding to the theoretically proposed interpersonal communication subdimension; Factor 3 mainly included items interpersonal interaction 9, 10, and 11, corresponding to the theoretically proposed relationship perception subdimension; and Factor 2 mainly comprised items interpersonal interaction 4, 7, 13, and 14, corresponding to the theoretically proposed willingness to interact subdimension. Except for the deleted items, all remaining items were retained and entered into subsequent analyses.

In summary, through item analysis and exploratory factor analysis conducted during the initial scale testing stage, this study systematically screened and optimized the initial item pool and factor structure, preliminarily establishing the formal structure of the *Healthy Psychological Effects of Physical Exercise Scale for College Students* and proceeding to the subsequent phase of confirmatory validation.

## 4 Validity and reliability testing of the formal healthy psychological effects of physical exercise scale for college students

### 4.1 Participants for the validity and reliability testing of the formal scale

To further examine the validity of the *Healthy Psychological Effects of Physical Exercise Scale for College Students*, a new sample of 945 participants was recruited for analysis, constituting Sample 1 of the present study. In addition, to further assess the test–retest reliability of the scale, a follow-up survey was conducted 1 month after the initial formal survey. Of the 945 participants in Sample 1, 597 provided valid follow-up responses, yielding a valid response rate of 63.17%; these participants constituted Sample 2. The distribution of demographic variables in Sample 2 was largely consistent with that of Sample 1, indicating good representativeness. The demographic characteristics of Sample 1 and Sample 2 are presented in Table 13.

**Table 13:** Summary of demographic characteristics for the formal scale testing (*N*_1_/*N*_2_ = 945/597).

Demographic variable	Category	Sample 1, *n* (%) or M (SD)	Sample 2, *n* (%) or M (SD)	Retained in sample 2 (%)
Gender	Male	392 (41.5)	276 (46.2)	70.4
	Female	553 (58.5)	321 (53.8)	58.0
Age	—	19.39 (1.34)	19.50 (1.38)	—
Place of family residence	Provincial capital city	205 (21.7)	123 (20.6)	60.0
	Prefecture-level city	182 (19.3)	112 (18.8)	61.5
	County-level city/town	272 (28.8)	182 (30.5)	66.9
	Township	108 (11.4)	64 (10.7)	59.3
	Rural area	178 (18.8)	116 (19.4)	65.2
Only-child status	Yes	376 (39.8)	244 (40.9)	64.9
	No	569 (60.2)	353 (59.1)	62.0
Grade level	Freshman	323 (34.2)	207 (34.7)	64.1
	Sophomore	292 (30.9)	179 (30.0)	61.3
	Junior	168 (17.8)	111 (18.6)	66.1
	Senior	162 (17.1)	100 (16.8)	61.7

Age is presented as mean (standard deviation), whereas categorical variables are presented as n (%). The “retained in sample 2 (%)” column indicates the proportion of participants in each Sample 1 category who provided valid follow-up responses.

### 4.2 Analysis strategy and statistical tools for the validity and reliability testing of the formal scale

First, descriptive statistics were used to summarize the demographic characteristics of the sample and the basic features of the scale variables. This approach helped to describe fundamental information such as gender and age distributions. Second, reliability analysis was conducted by calculating Cronbach's α, composite reliability (CR), and average variance extracted (AVE) to assess the internal consistency of the scale. Cronbach's α evaluates the consistency among items, CR reflects the reliability of the indicators, and AVE indicates the convergent validity of the constructs. Through these indices, the reliability of the scale was systematically evaluated. To verify the temporal stability and reliability of the scale, a test–retest procedure was conducted with a 1-month interval, and correlation analyses were performed among the subscales as well as between each subscale and the total score. Finally, confirmatory factor analysis (CFA) was employed to further examine the structural validity of the scale. Through CFA, the fit between the hypothesized factor structure and the observed data was evaluated using fit indices such as χ^2^*/*df, root mean square error of approximation (RMSEA), standardized root mean square residual (SRMR), comparative fit index (CFI), and Tucker–Lewis index (TLI), ensuring that the scale structure was robust and valid. In this study, Mplus 8.3 (Muthén & Muthén, Los Angeles, CA, USA) was used to conduct CFA. In addition, criterion-related validity was assessed by examining the correlations between the developed scale and external criteria. Taken together, these analytical strategies and statistical methods ensured a comprehensive evaluation of the reliability and validity of the scale and provided a solid empirical foundation for its subsequent application.

### 4.3 Reliability and convergent validity of the formal scale

In this study, to systematically evaluate the measurement quality of the formal scale, internal consistency was examined using Cronbach's α and composite reliability (CR), while convergent validity was assessed using average variance extracted (AVE). Generally, Cronbach's α and CR values greater than 0.70 indicate good internal consistency, whereas AVE values greater than 0.50 suggest that the construct explains a substantial proportion of the variance in its items and thus demonstrates adequate convergent validity. Taken together, these indices provide sufficient psychometric evidence to support the subsequent application and research use of the scale.

#### 4.3.1 Internal consistency and convergent validity of each subscale

Cronbach's α, composite reliability (CR), and average variance extracted (AVE) were calculated for the five subscales and their 15 subdimensions to comprehensively evaluate internal consistency and convergent validity. The results are summarized in Table 14.

**Table 14:** Cronbach's α, composite reliability, and average variance extracted for each subscale and subdimension.

Subscale	Subdimension	Cronbach's α	Composite reliability (CR)	Average variance extracted (AVE)
Emotional feelings	Psychological positive motivation	0.883	0.883	0.716
	Psychological calmness	0.889	0.889	0.728
	Emotional disturbance	0.931	0.931	0.693
Self-cognition	Competence efficacy	0.906	0.907	0.708
	Self-acceptance	0.876	0.877	0.705
	Body self-perception	0.889	0.889	0.727
Frustration coping	Concentration of energy	0.903	0.903	0.699
	Flexible thinking	0.873	0.873	0.697
	Optimistic mindset	0.918	0.918	0.736
Self-control and social adaptation efficacy	Academic coping	0.881	0.881	0.712
	Addiction control	0.895	0.895	0.681
	Campus adaptation	0.902	0.902	0.697
Interpersonal interaction	Interpersonal communication	0.915	0.915	0.730
	Relationship perception	0.883	0.883	0.717
	Willingness to interact	0.895	0.895	0.680

Table 14 presents the results of reliability and convergent validity for the 15 subdimensions across the five subscales. The Cronbach's α coefficients of the subdimensions ranged from 0.873 to 0.931, and the composite reliability (CR) values also ranged from 0.873 to 0.931. The average variance extracted (AVE) values ranged from 0.680 to 0.736. Overall, all subdimensions demonstrated good internal consistency and convergent validity, providing a reliable measurement foundation for subsequent analyses and applications. In addition, the internal consistency of the total scores for each subscale also reached satisfactory levels: Cronbach's α was 0.894 for the emotional feelings subscale, 0.886 for the self-cognition subscale, 0.880 for the frustration coping subscale, 0.861 for the self-control and social adaptation efficacy subscale, and 0.885 for the interpersonal interaction subscale. These results further support the high reliability of each subscale in terms of internal consistency.

#### 4.3.2 Test–retest reliability of each subscale

To examine the test–retest reliability of each subscale, 597 valid follow-up responses obtained 1 month after the initial formal survey were included in the test–retest reliability analysis. The specific results of the test–retest reliability analysis are presented in Table 15.

**Table 15:** Test–retest reliability of subdimensions and subscale total scores.

Subscale	Subdimension/total score	Test–retest reliability
Emotional feelings	Psychological positive motivation	0.707^***^
	Psychological calmness	0.731^***^
	Emotional disturbance	0.754^***^
	Emotional feelings (subscale)	0.722^***^
Self-cognition	Competence efficacy	0.769^***^
	Self-acceptance	0.765^***^
	Body self-perception	0.781^***^
	Self-cognition (subscale)	0.761^***^
Frustration coping	Concentration of energy	0.742^***^
	Flexible thinking	0.766^***^
	Optimistic mindset	0.756^***^
	Frustration coping (subscale)	0.771^***^
Self-control and social adaptation efficacy	Academic coping	0.724^***^
	Addiction control	0.709^***^
	Campus adaptation	0.706^***^
	Self-control and social adaptation efficacy (subscale)	0.686^***^
Interpersonal interaction	Interpersonal communication	0.714^***^
	Willingness to interact	0.726^***^
	Relationship perception	0.724^***^
	Interpersonal interaction (subscale)	0.711^***^

^***^p < 0.001.

Overall, the test–retest reliability coefficients obtained from the repeated measurement after 1 month were all above 0.65 and statistically significant, further demonstrating the reliability and temporal stability of the scale in measuring the relevant constructs. These results provide solid empirical support for the use of the scale in subsequent research and practical applications.

#### 4.3.3 Correlation analysis among subscales

To examine the correlations among the five subscales (emotional feelings, self-cognition, frustration coping, self-control and social adaptation efficacy, and interpersonal interaction), as well as their correlations with the total scale score, Pearson correlation analysis was conducted. The detailed results are presented in Table 16.

**Table 16:** Correlation analysis between each subscale and the total score.

Variable	Emotional feelings	Self-cognition	Frustration coping	Self-control and social adaptation efficacy	Interpersonal interaction	Total scale score
Emotional feelings	1					
Self-cognition	0.407^***^	1				
Frustration coping	0.489^***^	0.380^***^	1			
Self-control and social adaptation efficacy	0.488^***^	0.508^***^	0.561^***^	1		
Interpersonal interaction	0.478^***^	0.449^***^	0.425^***^	0.465^***^	1	
Total scale score	0.766^***^	0.717^***^	0.756^***^	0.778^***^	0.748^***^	1

^***^p < 0.001.

Pairwise correlations among the five subscales were all statistically significant (*r* = 0.380–0.561, all *p* < 0.001), indicating moderate positive correlations among the dimensions while suggesting that they remain relatively independent. The correlations between each subscale and the total scale score were relatively high (*r* = 0.717–0.778, all *p* < 0.001). Overall, all correlations among the subscales and between the subscales and the total score reached statistical significance, with most correlations ranging from moderate to high in magnitude. These findings support the assumption that the subscales assess related dimensions of the healthy psychological effects of physical exercise among college students, indicating a certain degree of internal consistency and structural validity among the subscales and between each subscale and the total score. Through this correlation analysis, it can be demonstrated that the subscales are meaningfully related to one another and are also highly correlated with the overall scale score, thereby providing evidence for the rationality of the scale's structure.

### 4.4 Validity testing of the formal scale

Based on the practical context of the present study and the applicability of different validity assessment methods, content validity, construct validity, and criterion-related validity were examined to evaluate the validity of the formal *Healthy Psychological Effects of Physical Exercise Scale for College Students*.

#### 4.4.1 Content validity

First, at the theoretical level, the development of the *Healthy Psychological Effects of Physical Exercise Scale for College Students* was grounded in an extensive review of domestic and international literature on the effects of physical exercise on college students' mental health. This ensured that the content of the scale comprehensively reflects the various aspects of the impact of physical exercise on college students' mental health. At the level of practical experience, data were collected on college students' actual experiences and perceptions of physical exercise through open-ended questionnaires and semi-structured interviews, providing an empirical foundation for the generation of scale items. Second, the scale items were systematically evaluated by researchers in related fields of college students' psychological benefits of physical exercise, including faculty members and doctoral students specializing in psychology and physical education. This process ensured an appropriate correspondence between the items and the psychological traits being measured, thereby meeting the requirements for content validity. Subsequently, doctoral students majoring in Chinese language and literature, along with a subset of scale respondents, were invited to evaluate the accuracy of item wording to ensure the clarity and comprehensibility of the scale items. Finally, the items were repeatedly revised and screened in light of China's sociocultural context and the current realities of physical exercise and academic development among Chinese college students, ensuring the appropriateness and representativeness of the scale content for this population. Taken together, the *Healthy Psychological Effects of Physical Exercise Scale for College Students* developed in this study demonstrates good content validity.

#### 4.4.2 Construct validity

In statistics, construct validity of a scale is typically examined using confirmatory factor analysis (CFA). In the present study, CFA was employed as the primary method to further examine the construct validity of the formal *Healthy Psychological Effects of Physical Exerci*se scale for college students.

To evaluate model fit, this study employed several commonly used fit indices along with their corresponding cutoff criteria (27, 28). Specifically, the χ^2^/df ratio was used to assess the overall fit between the model and the data, with values below 5 generally indicating an acceptable model fit. In addition, the root mean square error of approximation (RMSEA) was used to evaluate the balance between model complexity and error, with smaller values indicating better fit; values below 0.08 are typically considered indicative of good model fit with low approximation error. The standardized root mean square residual (SRMR) reflects the discrepancy between the model-predicted values and the observed data, with values below 0.06 regarded as evidence of good fit. In addition, the comparative fit index (CFI) and the Tucker–Lewis index (TLI) were used to evaluate the improvement of the proposed model relative to a null model. Values of these indices closer to 1 indicate better model fit, with values greater than 0.95 generally regarded as indicative of excellent fit. Taken together, these fit indices provide a comprehensive evaluation of the structural validity of the *Healthy Psychological Effects of Physical Exercise Scale for College Students*, ensuring a high degree of consistency between the proposed model and the observed data, and thereby offering empirical support for the construct validity of the scale.

**Figure 1:**
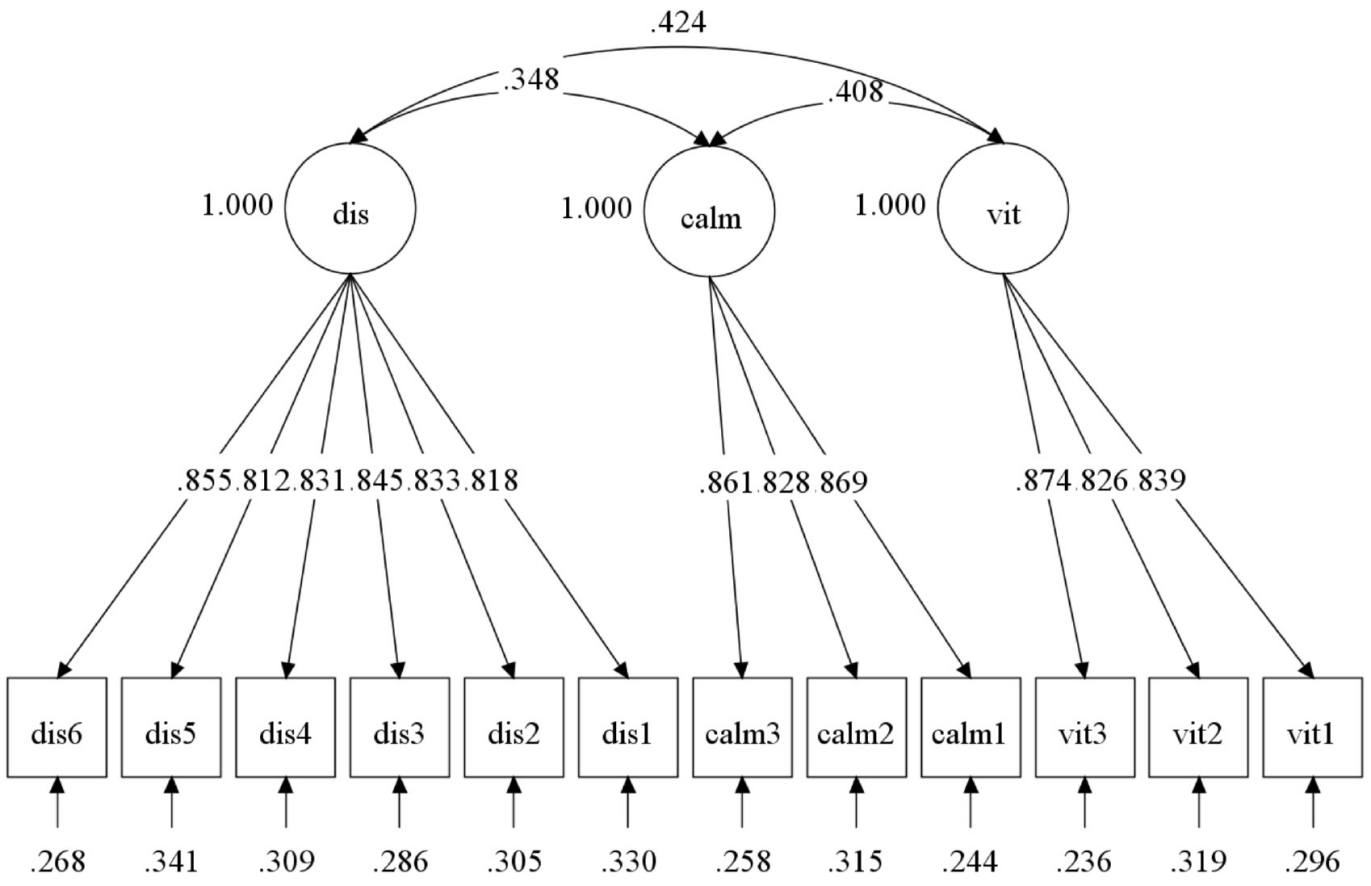
Factor structure model of the emotional feelings subscale. dis, emotional disturbance; calm, psychological calmness; vit, psychological positive motivation.

**Figure 2:**
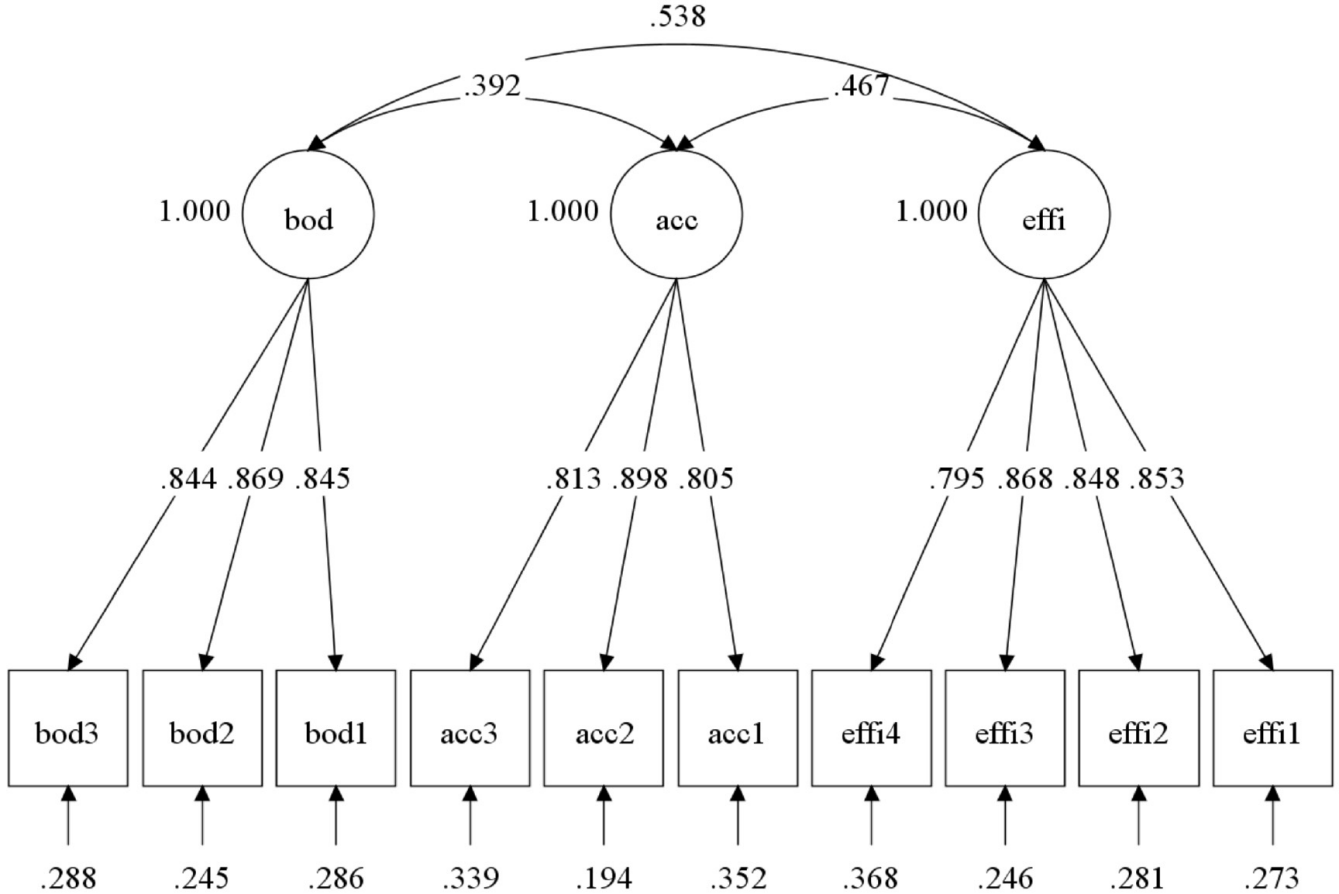
Factor structure model of the self-cognition subscale. bod, body self-perception; acc, self-acceptance; effi, competence efficacy.

**Figure 3:**
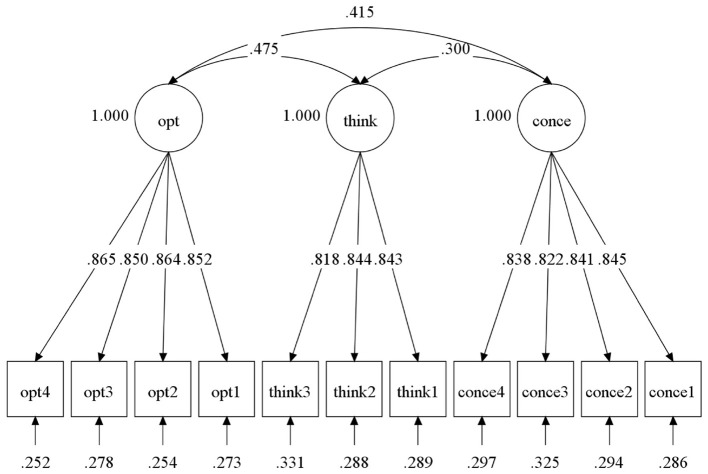
Factor structure model of the frustration coping subscale. opt, optimistic mindset; think, flexible thinking; conce, concentration of energy.

**Figure 4:**
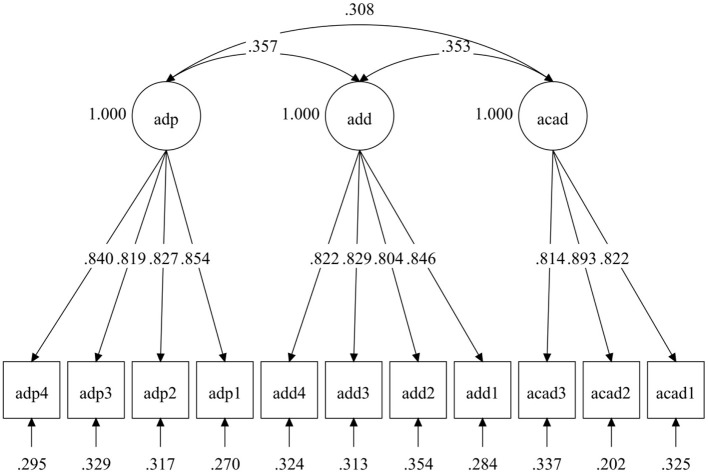
Factor structure model of the self-control and social adaptation efficacy subscale. adp, campus adaptation; add, addiction control; acad, academic coping.

**Figure 5:**
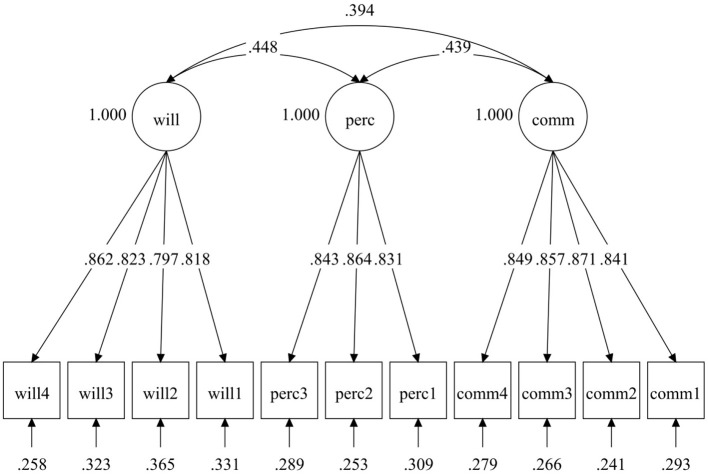
Factor structure model of the interpersonal interaction subscale. will, willingness to interact; perc, relationship perception; comm, interpersonal communication.

In this study, CFA was conducted separately for the hypothesized three-factor structures of the five subscales—emotional feelings, self-cognition, frustration coping, self-control and social adaptation efficacy, and interpersonal interaction. The corresponding model fit results are summarized in Table 17, and the factor structure diagrams for each subscale are presented in Figures 1–5.

**Table 17:** CFA fit indices for the five subscales.

Subscale	χ^2^/df	CFI	TLI	RMSEA	SRMR
Emotional feelings	4.391	0.978	0.971	0.060	0.021
Self-cognition	3.213	0.988	0.984	0.048	0.016
Frustration coping	3.556	0.985	0.980	0.052	0.018
Self-control and social adaptation efficacy	2.978	0.987	0.983	0.046	0.019
Interpersonal interaction	3.345	0.986	0.981	0.050	0.016

Cutoff values: χ^2^/df < 5.000, CFI > 0.950, TLI > 0.950, RMSEA < 0.080, SRMR < 0.060.

Based on the results of the confirmatory factor analyses, all five subscales demonstrated good model fit, with all fit indices falling within acceptable ranges. In terms of overall fit, the χ^2^/df values for the subscales ranged from 2.978 to 4.391, all below the commonly accepted criterion of 5.000, indicating an acceptable level of overall model–data fit. Meanwhile, RMSEA values ranged from 0.046 to 0.060, well below the conventional threshold of 0.080, and SRMR values ranged from 0.016 to 0.021, substantially lower than the commonly used cutoff of 0.060. These results indicate small model residuals and suggest that the models were able to adequately reproduce the sample covariance structure. With respect to incremental fit, CFI and TLI values ranged from 0.978 to 0.988 and from 0.971 to 0.984, respectively. All values exceeded the acceptable standard of 0.900 and generally surpassed the more stringent criterion of 0.950, further indicating that the proposed models showed strong explanatory power and superior fit relative to the independence models. Taken together, across different fit index categories—including absolute fit, residual-based fit, and incremental fit—the CFA results for the five subscales were consistently satisfactory. These findings provide robust evidence supporting the hypothesized three-factor structures of each subscale and, in turn, support the structural validity of the subscales of the *Healthy Psychological Effects of Physical Exercise Scale for College Students*.

**Figure 6:**
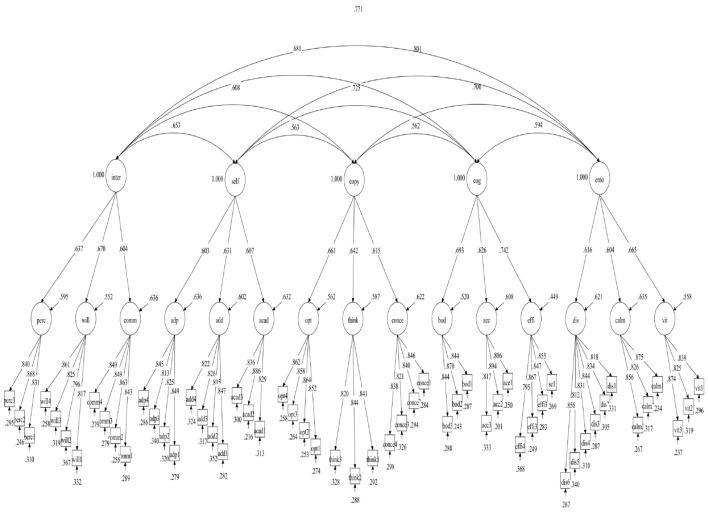
Factor structure model of the overall scale. emo, emotional feelings; cog, self-cognition; copy, frustration coping; self, self-control and social adaptation efficacy; inter, interpersonal interaction. All other specifications are consistent with those reported in the previous analyses.

Building on the separate verification of the hypothesized three-factor structures of the five subscales, a confirmatory factor analysis (CFA) was further conducted on the overall scale model to examine the rationality and goodness of fit of the scale's overall structure. The results indicated that the overall model demonstrated good fit, with all fit indices falling within acceptable ranges. Specifically, the χ^2^/df value was 3.037, which is below the cutoff criterion of 5.000, indicating a satisfactory level of model–data fit and meeting the standards for confirmatory factor analysis. The RMSEA value was 0.046, lower than the threshold of 0.080, suggesting a small approximation error and an overall favorable model fit. The SRMR value was 0.041, well below 0.060, indicating minimal discrepancies between model-predicted and observed values and an excellent fit. In terms of incremental fit indices, the CFI value was 0.925 and the TLI value was 0.921, both exceeding the cutoff criterion of 0.900 (28), indicating that the model exhibited good fit. The factor structure model of the overall scale is shown in Figure 6.

Overall, the primary fit indices of the model (χ^2^/df, RMSEA, SRMR, CFI, and TLI) all demonstrated satisfactory fit and met the criteria for confirmatory factor analysis, thereby providing evidence for the sound structural validity of the scale.

#### 4.4.3 Criterion-related validity

To further examine the criterion-related validity of the *Healthy Psychological Effects of Physical Exercise Scale for College Students*, this study employed criterion validity testing by using the *Positive Mental Characters Scale for Chinese College Students* and the *Chinese Adult Mental Health Scale* as external criteria reflecting comprehensive positive psychological factors and negative psychological factors, respectively (29, 30). Among these instruments, the *Positive Mental Characters Scale for Chinese College Students* is designed for the college student population and is capable of comprehensively reflecting students' levels of positive psychological qualities and psychological resources. It is theoretically expected to be positively associated with the emphasis of the present scale on the enhancement of positive experiences and adaptive psychological functioning. This scale contains 62 items, and in the present study, the total scale demonstrated a Cronbach's α of 0.967, indicating extremely high internal consistency and providing a stable reference for subsequent validity testing. In addition, the *Chinese Adult Mental Health Scale* reflects levels of negative psychological states and psychological distress and can therefore serve as an external criterion for the negative-alleviation dimension, for which a negative association with the scores of the present scale is theoretically expected. This scale comprises 80 items, and in the present study, the total scale yielded a Cronbach's α of 0.973, indicating extremely high internal consistency and supporting its use for subsequent validity testing. Based on these considerations, correlation analyses were conducted between each subscale and its subdimensions of the present scale and the scores of the two criterion measures. The results are presented in Table 18.

**Table 18:** Criterion-related validity of each subscale.

Subscale	Subdimension/total score	Positive psychology	Negative psychology
Emotional feelings	Psychological positive motivation	0.281^***^	−0.318^***^
	Psychological calmness	0.296^***^	−0.287^***^
	Emotional disturbance	0.274^***^	−0.287^***^
	Emotional feelings (subscale)	0.375^***^	−0.394^***^
Self-cognition	Competence efficacy	0.273^***^	−0.281^***^
	Self-acceptance	0.282^***^	−0.300^***^
	Body self-perception	0.287^***^	−0.334^***^
	Self-cognition (subscale)	0.359^***^	−0.390^***^
Frustration coping	Concentration of energy	0.259^***^	−0.303^***^
	Flexible thinking	0.292^***^	−0.259^***^
	Optimistic mindset	0.305^***^	−0.327^***^
	Frustration coping (subscale)	0.383^***^	−0.398^***^
Self-control and social adaptation efficacy	Academic coping	0.245^***^	−0.285^***^
	Addiction control	0.266^***^	−0.233^***^
	Campus adaptation	0.270^***^	−0.318^***^
	Self-control and social adaptation efficacy (subscale)	0.356^***^	−0.382^***^
Interpersonal interaction	Interpersonal communication	0.283^***^	−0.341^***^
	Willingness to interact	0.229^***^	−0.292^***^
	Relationship perception	0.240^***^	−0.224^***^
	Interpersonal interaction (subscale)	0.326^***^	−0.371^***^

^***^p < 0.001.

As shown in Table 18, all subdimensions and the total scores of the five subscales were significantly positively correlated with the positive psychological indicators (*r* = 0.229–0.383, *p* < 0.001) and significantly negatively correlated with the negative psychological indicators (*r* = −0.224 to −0.398, *p* < 0.001). The directions of these correlations were consistent with the theoretical expectations, indicating that the present scale is capable of simultaneously capturing the enhancement of positive psychological experiences and functions as well as the alleviation of negative psychological states following physical exercise. Notably, the total subscale scores exhibited relatively stronger correlations with both types of external criteria (positive psychology: *r* = 0.326–0.383; negative psychology: *r* = −0.371 to −0.398), suggesting that the composite scores are more representative in reflecting overall healthy psychological effects. Overall, these findings indicate that the five subscales demonstrate stable and consistent associations in distinguishing between positive and negative psychological states, thereby providing empirical support for the criterion-related validity of the *Healthy Psychological Effects of Physical Exercise Scale for College Students*.

## 5 Discussion

The results of the present study provide empirical support for the preliminary dimensional framework proposed before scale construction. All five subscales—emotional feelings, self-cognition, frustration coping, self-control and social adaptation efficacy, and interpersonal interaction—along with their corresponding 15 subdimensions, were retained in the final scale and showed clear and stable structural patterns in both exploratory and confirmatory factor analyses. The items of the final scale are provided in Supplementary Table 1. These findings are consistent with the initial theoretical assumptions and suggest that the proposed framework can effectively represent the multidimensional healthy psychological effects of physical exercise among college students. Furthermore, the CFA fit indices for the five subscales were overall at satisfactory levels, supporting the rationality of their hypothesized three-factor structures. The CFA of the overall scale also met commonly accepted criteria, indicating that the overall factor structure was able to adequately reproduce the sample covariance structure. In terms of reliability, the indices of internal consistency, composite reliability, and convergent validity for each dimension demonstrated generally good performance, and the test–retest results further indicated that the scale maintained stable consistency over a certain time interval. With respect to criterion-related validity, the scale showed positive correlations with positive psychological indicators and negative correlations with negative psychological indicators, with correlation directions consistent with theoretical expectations. These findings indicate that the scale is capable of simultaneously capturing the comprehensive psychological outcomes of both “positive enhancement” and “negative alleviation” following physical exercise among college students. Moreover, based on the evidence supporting the stability of the scale structure as well as its reliability and validity, the scale maintains a clear contextual focus on psychological changes associated with physical exercise while allowing flexibility in its temporal frame of reference. The wording of the items can be adapted according to different research designs, enabling the scale to assess not only immediate psychological changes following exercise, but also stage-specific or longitudinal changes over time. In intervention studies, it may also serve as a pretest instrument to characterize participants' relevant psychological baseline status within a unified measurement framework. This methodological feature broadens the applicability of the scale and supports its use across different research paradigms, including cross-sectional studies and intervention studies. Overall, these lines of evidence jointly support that the *Healthy Psychological Effects of Physical Exercise Scale for College Students* demonstrates good structural validity, internal consistency, and external criterion-related validity, and can be used as a quantitative measurement tool in subsequent research and practical evaluations.

From the specific content of the *Healthy Psychological Effects of Physical Exercise Scale for College Students*, this scale represents the first comprehensive assessment tool specifically focused on the healthy psychological effects of physical exercise among the college student population. In the present study, the structural validity of all five subscales was supported, indicating that psychological changes following physical exercise are not characterized by linear improvement along a single dimension. Within the Chinese college student population, students commonly experience pressures from multiple sources in their academic and daily lives and tend to exhibit pronounced emotional fluctuations (31). The configuration of the emotional feelings subscale is consistent with the overall definition of the healthy psychological effects of physical exercise adopted in this study, in that it conceptualizes post-exercise subjective cognitive and emotional responses conducive to mental health development as a comprehensive psychological outcome. The three-factor structure of this subscale was supported by factor analyses, and its fit indices and reliability performance indicate that it can stably distinguish different types of emotional changes following physical exercise. In particular, it highlights the dual effects of physical exercise on college students' emotional functioning: on the one hand, promoting the growth of positive experiences such as enthusiasm, vitality, enjoyment, ease, and calmness; and on the other hand, reducing negative emotions and emotional distress such as depression, low mood, restlessness, and inner emptiness. Compared with existing emotion measurement instruments, this subscale both aligns with and extends prior approaches in terms of measurement orientation and content coverage. First, classic instruments such as the POMS are designed to assess general changes in individuals' mood states (32), encompassing multiple affective dimensions such as tension, depression, anger, vigor, fatigue, and confusion. However, their original purpose was not to be structurally constructed around the specific context of “post–physical exercise.” As a result, when applied to the interpretation of exercise-related emotional benefits, they tend to remain at the level of descriptive mood states rather than capturing the distinctive emotional changes associated with physical exercise (33). Second, the PANAS primarily assesses emotional experiences along two dimensions—positive affect and negative affect (34)—but offers relatively limited differentiation of the low-arousal restorative experiences commonly observed after exercise, such as calmness and relaxation. In contrast, the emotional feelings subscale developed in this study simultaneously incorporates the activation of high-arousal positive experiences in the exercise context, such as enthusiasm and vitality, as well as the restoration of low-arousal positive experiences, such as ease and calmness, while also assessing the alleviation of negative emotions and emotional distress. In doing so, it provides a more comprehensive representation of the emotional-level healthy psychological effects of physical exercise under a health-promotion orientation.

For college students who are at a developmental stage characterized by identity formation and heightened sensitivity in self-evaluation, the positive changes brought about by physical exercise in terms of competence experiences, bodily sensations, and perceptions of appearance are more likely to facilitate a renewed affirmation of self-ability and self-worth (35). Therefore, it is necessary to conduct structured measurement from the perspective of self-cognition. The self-cognition subscale is designed to assess post-exercise changes in self-cognition based on college students' subjective understanding and evaluation of the self, and establishes a measurement framework encompassing three aspects: competence beliefs, value affirmation, and body perception. Its three-factor structure was supported by factor analysis. With regard to existing self-related measurement instruments, the Rosenberg Self-Esteem Scale is primarily used to assess individuals' relatively stable global self-esteem (36), and its items are more suitable for trait-level self-evaluation rather than context-specific gains following physical exercise. General self-efficacy scales emphasize generalized beliefs about competence in dealing with various pressures or tasks (37), but they often lack sufficiently fine-grained and structured differentiation for capturing specific changes in self-cognition induced by physical exercise, such as those arising from bodily energy experiences and enhanced perceptions of competence (38, 39). In addition, body-related self-measures such as the physical self-perception profile place greater emphasis on individuals' perceptions of sport competence, physical attractiveness, and physical worth (40), with their core focus typically centered on the physical domain of self-concept. These instruments do not integrate bodily self-perceptions with general competence beliefs and self-worth affirmation into a comprehensive framework of “post-exercise healthy psychological outcomes.” In contrast, the self-cognition subscale developed in this study simultaneously encompasses changes in three key cognitive dimensions following physical exercise—competence efficacy, self-acceptance, and body self-perception. It not only retains the core concerns of ability and value judgments emphasized in self-esteem and self-efficacy research, but also incorporates bodily experiences and perceptions of physical appearance as important cognitive changes after physical exercise. In doing so, it more closely aligns with the actual ways in which the self-system changes under a health-promotion orientation of physical exercise, and provides a more comprehensive representation of the positive effects of physical exercise on college students' self-evaluation and self-affirmation within a health-promotion framework.

In addition, within the realistic context of Chinese college students—characterized by intensive academic demands, frequent role transitions, and diverse sources of stress—the psychological resources provided by physical exercise are often manifested not only as improvements in emotional experiences but also externalized as higher levels of task focus, greater cognitive flexibility, and more positive attributions and expectations. These changes, in turn, facilitate a shift from passive endurance to active regulation when students confront stressful situations (41). In other words, individuals may undergo a functional transition from passive coping to proactive adjustment under stress. The key question, however, is whether summarizing post-exercise stress-adaptation benefits solely in terms of emotional improvement obscures more critical process-oriented capacities, such as sustained attention, cognitive shifting, and positive persistence. Based on this consideration, the frustration coping subscale constructs its measurement framework around three subdimensions—Concentration of Energy, Flexible Thinking, and Optimistic Mindset—which, respectively correspond to post-exercise enhancements in the ability to maintain focus and control emotional interference, strengthened capacities for cognitive reappraisal and multi-perspective thinking, and the consolidation of positive expectations and action motivation when facing adversity. In other words, within the scope of what is measured by this subscale, emotion is not excluded; rather, it is repositioned within a functional optimization chain characterized by greater emotional regulability, more stable attention, more flexible thinking, and more sustained action. In this way, the subscale more closely reflects the characteristics of psychological functional optimization in frustration coping from a health-promotion perspective of physical exercise. The three-factor structure of this subscale was supported by factor analysis, and its fit indices and reliability performance indicate that it can stably differentiate multiple dimensions of psychological adaptation when college students face stress and setbacks after physical exercise. Compared with existing measures of frustration coping or psychological resilience, this subscale both aligns with and extends prior instruments in terms of measurement anchors and the integration of content. First, instruments such as the COPE and Brief COPE focus on categorizing the coping strategies individuals adopt in stressful situations (42, 43). However, their items are typically framed around general stress events, which makes them prone to remaining at the level of strategy enumeration and limits their ability to capture exercise-related functional gains. Second, resilience measures such as the Connor–Davidson resilience scale emphasize relatively stable trait-like capacities for maintaining adaptation and recovery in the face of adversity (44). Their measurement orientation is therefore biased toward trait-level resilience rather than being contingent on post-exercise experiential changes, making them more suitable for predicting who is more likely to persist or recover, rather than for assessing functional changes following physical exercise. Third, the cognitive emotion regulation questionnaire (CERQ) emphasizes individuals' cognitive strategies when confronting negative events, but its content focuses primarily on measuring *how* individuals think after adverse experiences. As a result, it provides relatively limited coverage of functional changes commonly observed after physical exercise, such as the restoration of attentional resources and improvements in task-focused engagement (45). Fourth, optimism, as an important psychological resource, is typically measured using the life orientation test (LOT) or the revised version (LOT-R), which assess relatively stable life orientations (46, 47). However, these instruments are likewise oriented toward trait-level evaluation and have limited capacity to directly capture state-like enhancements following physical exercise, such as being more willing to persist and having greater confidence in one's ability to resolve difficulties in stressful situations. In contrast, the frustration coping subscale developed in this study integrates into a single framework three core components that are more readily experienced by college students after physical exercise and more directly associated with stress-adaptation performance. Specifically, concentration of energy emphasizes the ability to reduce emotional interference and maintain task-focused attention in stressful situations; flexible thinking captures the tendency and capacity to adjust perspectives and engage in cognitive reappraisal when confronting setbacks; and optimistic mindset reflects the motivation to maintain positive expectations and sustain engagement in problem solving under adversity. This structure not only resonates with prior coping research highlighting the importance of attentional control, cognitive reappraisal, and positive expectations in adaptive functioning, but also enhances, at the measurement level, the orientation and structural comparability of healthy psychological effects of physical exercise. Consequently, it is better suited for evaluating the facilitative effects of physical exercise on frustration coping functioning among college students.

Moreover, within the Chinese educational context, the challenges that college students most commonly encounter in their academic and daily lives are often characterized by high task demands, numerous external temptations, and rapid role adaptation (48). Physical exercise may therefore be further externalized as stronger self-control, clearer goal management, and more positive integration into campus life (49, 50). If these forms of “externalization” are understood as key manifestations of exercise effects, the scale must further assess whether, and in what ways, college students' self-regulation and adaptation efficacy change across three high-frequency contexts: academic tasks, media-related temptations, and campus life. Accordingly, the self-control and social adaptation efficacy subscale organizes its measurement content around three subdimensions—academic coping, addiction control, and campus adaptation. academic coping corresponds to post-exercise improvements in learning engagement and time-management efficacy; addiction control refers to enhanced impulse inhibition and behavioral self-discipline in the face of immediate-gratification temptations such as short-video platforms, social media, and online games; and campus adaptation focuses on the optimization of adaptive functioning in areas such as resource utilization, activity participation, balance between academic and daily life, and the construction of a sense of belonging. The advantage of this structure is that it brings self-control and adaptation back from abstract personality traits into the lived world of college students, making the situational framing of items more concrete and the changes more empirically observable. This orientation also becomes clearer when contrasted with existing measurement instruments. In existing self-control research, commonly used self-control scales or brief self-control scales are primarily designed to assess relatively stable, trait-like self-control capacities (51). Their items are typically not framed with post-exercise experiential change as a measurement premise and are therefore more suitable for reflecting “what kind of person I usually am.” In the domain of digital behavior management, scales assessing internet addiction or problematic mobile phone use are mostly intended for screening and evaluating addiction risk or severity (52), emphasizing compulsive use, withdrawal symptoms, and functional impairment, and are thus well-suited for risk identification. However, when the research focus is on more constructive regulatory processes following physical exercise—such as reduced impulsivity, restoration of attentional resources, and increased engagement in real-world activities—a problem-screening orientation does not necessarily align well with a health-promotion–oriented explanatory framework. In addition, classic college adjustment scales in the field of campus adaptation typically adopt frameworks encompassing academic adjustment, social adjustment, personal–emotional adjustment, and institutional attachment, enabling systematic assessment of students' adaptation to the new university environment (53). However, these instruments are likewise oriented toward relatively broad issues of college entry and adjustment and do not specifically focus on improvements in adaptive functioning driven by changes in psychological resources within the context of physical exercise. Consequently, when they are used to explain “exercise-related benefits in campus integration,” a mismatch may arise between the measurement context and the explanatory target. In contrast, the self-control and social adaptation efficacy subscale developed in this study integrates three types of real-life challenges that are most representative for college students into a single structural framework. The three-factor structure of this subscale was supported by factor analysis, and its fit indices and reliability performance indicate that it can stably distinguish different facets of change related to self-regulation and environmental integration following physical exercise among college students. On the one hand, in terms of content, it retains the core concerns of self-control research regarding impulse inhibition and behavioral regulation, while concretizing these concerns in relation to high-frequency digital self-regulation issues faced by college students. In this way, “addiction control” is rendered more closely aligned with the behavioral ecology of contemporary campus life. On the other hand, the academic coping dimension emphasizes changes that can be directly experienced by individuals, such as learning attitudes, task-focused engagement, and time-management efficacy, allowing exercise-related benefits to correspond more directly to functional performance in academic stress contexts. Meanwhile, the campus adaptation dimension integrates key components such as resource utilization, extracurricular participation, balance between academic and daily life, and the construction of a sense of belonging, thereby conceptualizing “adaptation” not merely as reduced stress or improved mood, but as a stronger emphasis on individuals' post-exercise proactivity and sense of competence in transforming the campus environment into a context that supports their personal development.

Compared with the immediate fluctuations of emotional experience, college students' mental health and adaptive functioning are also profoundly influenced by the quality of social connections, with needs for belonging and social support widely regarded as fundamental foundations for maintaining good psychological functioning (54). In this regard, physical exercise may not only facilitate the activation of social resources through shared activities, opportunities for interaction, and a sense of group belonging (55), but also foster greater confidence in interpersonal interactions, enhanced feelings of relational security, and more proactive tendencies toward social engagement. In this way, physical exercise can provide important support for college students' social adaptation (56, 57). Accordingly, the interpersonal interaction subscale in the present study constructs its measurement framework around three subdimensions—interpersonal communication, relationship perception, and willingness to interact—covering, respectively, overt communication competence, internal relational cognitive schemas, and latent prosocial motivation. The three-factor structure of this subscale was supported by factor analysis, and its fit indices and reliability performance indicate that it can stably differentiate different facets of change in social psychological functioning among college students following physical exercise. Within the structural framework of the present study, two practical social concerns faced by college students—“whether I can communicate more effectively” and “whether I am willing to approach others”—are incorporated simultaneously and are, respectively represented by the Interpersonal Communication and willingness to interact subdimensions. In contrast, relationship perception retains key focal points from research on social support and social connectedness, such as perceived support, trust, and belonging, and further operationalizes them as enhanced perceptions of support availability and experiences of friendliness and care, along with reduced sensitivity to others' evaluations. In doing so, this subdimension more closely aligns with the real needs of college students in contexts characterized by social pressure and concerns about self-presentation. Compared with existing measures of social support or interpersonal functioning, perceived social support instruments—such as the multidimensional scale of perceived social support—primarily assess perceived support from family, friends, and significant others (58). However, their items tend to focus on static evaluations of “existing support” and seldom directly address process-oriented changes following physical exercise, such as improvements in communication efficacy or enhanced motivation for social engagement. In addition, measures of social connectedness or belonging can capture individuals' subjective feelings of closeness and belonging with others (59), but when applied to explaining post-exercise improvements in social adaptation, they often remain at the level of general connectedness descriptions. Furthermore, interpersonal competence–oriented instruments, such as interpersonal competence questionnaires, emphasize social skill structures including relationship initiation, conflict management, self-disclosure, and emotional support (60). However, their measurement anchors are generally grounded in abilities or tendencies in everyday interpersonal contexts, without an explicit specification of the physical exercise context, thereby limiting their capacity to capture interpersonal psychological changes associated with physical exercise.

From the perspective of the overall scale, this study operationalizes post-exercise psychological changes as a comprehensive construct and develops a more targeted and ecologically valid *Healthy Psychological Effects of Physical Exercise Scale for College Students*. This approach aligns with the mechanism by which physical exercise may influence psychological states through multiple pathways, including emotional regulation, self-efficacy, behavioral self-control, and social connectedness. The five subscales together achieve a continuous extension in content coverage—from emotional experiences to self-cognition, from stress coping to self-regulation and social adaptation, and further to interpersonal connectedness. In this way, the scale is able to capture both domain-specific psychological changes following physical exercise and, under a shared measurement anchor and health-promotion orientation, to form an integrated and coherent structural system. Its advantages lie not only in simultaneously accounting for positive enhancement and negative alleviation, but also in organizing measurement content around the developmental tasks and exercise contexts of Chinese college students, thereby ensuring both theoretical traceability and strong ecological relevance, as well as enhanced comparability across studies. It is precisely on the basis of this structure that the proposal and use of the overall scale acquire clearer significance, as it can provide an integrated representation of psychological changes across multiple domains following physical exercise within a unified measurement framework. It not only preserves the ability of each subscale to distinguish changes in specific psychological domains, but also provides a comprehensive outcome indicator for subsequent related research, thereby enhancing the completeness and evidential comparability of measuring the healthy psychological effects of physical exercise. Compared with traditional comprehensive mental health measurement instruments such as the SCL-90, which primarily focus on negative psychological symptoms such as depression and anxiety, the present scale is no longer confined to a single pathological perspective. Instead, it adopts a holistic, health-oriented measurement approach by conceptualizing healthy psychological effects as a comprehensive psychological outcome that simultaneously encompasses the enhancement of positive experiences and the alleviation of negative states. In contrast to subsequently developed comprehensive instruments that focus on assessing general positive psychological qualities—such as the positive mental characters scale for Chinese school students (61)—a salient feature of the present scale lies in its context specificity. The scale focuses on measuring relevant psychological changes manifested by college students in the context of physical exercise, thereby distinguishing it from instruments that primarily assess relatively stable positive psychological qualities. This makes the measurement more closely aligned with the actual formation process of exercise-related psychological outcomes and avoids inferring exercise effects merely from differences in stable psychological traits. Moreover, compared with existing scales of the psychological benefits of physical exercise that primarily target primary and secondary school students or adolescent populations, the present scale achieves more precise developmental-stage alignment. The inclusion of subdimensions such as academic coping, campus adaptation, addiction control, relationship perception, and willingness to interact directly responds to the specific developmental tasks and psychological needs of Chinese college students in domains including academic management, self-identity, social integration, and future planning. Accordingly, the item design and situational framing of the scale are better tailored to the real-life context of university life. Within an integrated framework, the scale systematically covers multidimensional psychological constructs spanning emotion, cognition, behavior, and social adaptation, enabling each dimension to effectively capture typical psychological changes that college students may experience after physical exercise, thereby enhancing the ecological validity and population applicability of the measurement.

It should be noted that the present scale is not a mental health screening instrument in the general sense, nor is it intended to replace existing specialized measures of depression, anxiety, psychological symptoms, or positive psychological qualities. The key difference between the present scale and general mental health questionnaires lies in their assessment focus. General mental health questionnaires usually describe students' overall psychological condition or relatively stable psychological characteristics, whereas the present scale focuses on healthy psychological changes that students perceive in relation to physical exercise. Therefore, a student with good general mental health may not necessarily report equally strong exercise-related psychological changes. Such a student may already have relatively stable emotional regulation, sufficient social support, or effective coping resources, so the additional changes perceived in relation to physical exercise may be less salient. Conversely, students who rely more on physical exercise for emotional regulation, self-confidence, stress coping, or social connection may report more noticeable exercise-related psychological effects, even when their general mental health level is not particularly high. In this sense, the present scale focuses on examining the healthy psychological changes that students perceive in relation to physical exercise, rather than simply judging their general mental health status. Its primary value lies in providing a comprehensive psychological health outcome indicator for research on physical exercise, physical activity, and related health promotion, thereby simultaneously reflecting the enhancement of positive psychological experiences, the improvement of adaptive psychological functioning, and the alleviation of negative psychological states. At the same time, the temporal frame of reference for the scale items can be aligned with the study design and measurement purpose, as the selection of recall periods in self-report outcome measures should consider the study design, the nature of the construct, and the intended use of the outcome measure (62). With appropriate temporal anchors, the scale may be used to assess state-like psychological changes following a single session or short-term exercise (63), and it may also be combined with temporal anchors such as “compared with the previous month” to track psychological changes after continuous exercise or stage-based interventions. Future research is still needed to further examine its measurement sensitivity and cross-context applicability across different exercise cycles, exercise modalities, and sample populations.

Overall, the *Healthy Psychological Effects of Physical Exercise Scale for College Students* developed in this study is a comprehensive measurement instrument with a clear structure and sound reliability and validity. It covers key psychological domains, including emotional feelings, self-cognition, frustration coping, self-control and social adaptation efficacy, and interpersonal interaction, and can systematically assess comprehensive mental health outcomes manifested by college students after physical exercise or related health promotion activities, including the enhancement of positive psychological experiences, the improvement of adaptive psychological functioning, and the alleviation of negative psychological states. The development of this instrument enriches the available approaches for measuring the healthy psychological effects of physical exercise among college students and provides a more consistent measurement foundation for future research to quantitatively examine the associations between physical exercise behaviors and comprehensive mental health levels among college students, as well as their underlying mechanisms.

## 6 Limitations and future directions

This study also has several limitations. First, although the scale demonstrated good reliability and validity in the present research, the sample was drawn exclusively from Chinese college students, which may limit the generalizability of the findings to other cultural or educational contexts. Future studies could examine the cross-cultural applicability of the scale through translation, tests of measurement invariance, and validation in more diverse student populations. Second, the validity evidence in this study relied primarily on self-report measures, which may be influenced by social desirability or response biases. Future research could incorporate multi-method approaches, such as behavioral indicators and physiological measures, to further strengthen the construct validity of the scale. Finally, the present study focused on the overall psychometric properties of the scale and did not examine its sensitivity to different types, intensities, or durations of physical exercise. Future research may use this scale to explore how different exercise modalities or intervention designs are differentially associated with various dimensions of healthy psychological effects.

## 7 Conclusions

This study developed and validated the healthy psychological effects of physical exercise scale for college students. The scale shows a clear multidimensional structure and satisfactory reliability and validity, and is able to capture comprehensive healthy psychological changes following physical exercise, including positive psychological enhancement, improvements in adaptive psychological functioning, and the alleviation of negative psychological states. By focusing on post-exercise psychological changes within the developmental and situational context of college students, the scale provides a practical and psychometrically sound tool for future research and applied evaluations of exercise-related mental health outcomes.

## Data Availability

The raw data supporting the conclusions of this article will be made available by the authors, without undue reservation.
