# Supplementary material for: Development and validation of a scale of healthy psychological effects of physical exercise among Chinese college students from a multidimensional mental health perspective
Source: Front Public Health. 2026 Jun 26;14:1866486. doi: 10.3389/fpubh.2026.1866486 (PMC13352479; doi:10.3389/fpubh.2026.1866486)
Supplement: Supplementary file 1 [file Table_1.doc]

| *Healthy Psychological Effects of Physical Exercise Scale for College Students* | | | | | |
| --- | --- | --- | --- | --- | --- |
| After engaging in physical exercise, I feel that… | | | | | |
| Items | Strongly Disagree | Disagree | Neutral | Agree | Strongly Agree |
| 1. I feel more enthusiastic. | 1 | 2 | 3 | 4 | 5 |
| 2. I feel more vibrant. | 1 | 2 | 3 | 4 | 5 |
| 3. My life feels more enjoyable. | 1 | 2 | 3 | 4 | 5 |
| 4. I feel more relaxed and at ease. | 1 | 2 | 3 | 4 | 5 |
| 5. My emotions feel more stable. | 1 | 2 | 3 | 4 | 5 |
| 6. I experience greater inner calmness and harmony. | 1 | 2 | 3 | 4 | 5 |
| 7. I feel happier and more comfortable. | 1 | 2 | 3 | 4 | 5 |
| 8. Feelings of inner suppression have gradually decreased. | 1 | 2 | 3 | 4 | 5 |
| 9. My mood feels less low than before. | 1 | 2 | 3 | 4 | 5 |
| 10. Feelings of inner emptiness have diminished. | 1 | 2 | 3 | 4 | 5 |
| 11. Feelings of restlessness have decreased. | 1 | 2 | 3 | 4 | 5 |
| 12. My daily life feels more fulfilling. | 1 | 2 | 3 | 4 | 5 |
| 13. I am confident about achieving success in life. | 1 | 2 | 3 | 4 | 5 |
| 14. I feel more energized to handle things. | 1 | 2 | 3 | 4 | 5 |
| 15. I feel more confident in my abilities. | 1 | 2 | 3 | 4 | 5 |
| 16. I believe that my personal development should not be easily influenced by others’ behaviors or opinions. | 1 | 2 | 3 | 4 | 5 |
| 17. I am better able to recognize my unique value and strengths. | 1 | 2 | 3 | 4 | 5 |
| 18. I believe that I have many strengths and positive qualities. | 1 | 2 | 3 | 4 | 5 |
| 19. I feel that I am more attractive. | 1 | 2 | 3 | 4 | 5 |
| 20. feel that my limbs are stronger. | 1 | 2 | 3 | 4 | 5 |
| 21. I feel that my physical stamina is greater. | 1 | 2 | 3 | 4 | 5 |
| 22. I feel more satisfied with my physical appearance. | 1 | 2 | 3 | 4 | 5 |
| 23. I am able to exclude other distractions and focus my attention more effectively on the challenges at hand. | 1 | 2 | 3 | 4 | 5 |
| 24. I am able to think more clearly when facing pressure. | 1 | 2 | 3 | 4 | 5 |
| 25. I am better able to manage uncomfortable emotions in a reasonable way. | 1 | 2 | 3 | 4 | 5 |
| 26. I can focus my attention more on the current problem rather than avoiding it. | 1 | 2 | 3 | 4 | 5 |
| 27. When I need to break through a bottleneck, I am better able to step outside fixed patterns of thinking. | 1 | 2 | 3 | 4 | 5 |
| 28. I am more willing to change my perspective on problems to make myself feel better. | 1 | 2 | 3 | 4 | 5 |
| 29. When facing difficulties, I am able to maintain a calm state of mind. | 1 | 2 | 3 | 4 | 5 |
| 30. I am better able to see the positive side of things. | 1 | 2 | 3 | 4 | 5 |
| 31. I am confident that I can find ways to cope with current difficulties. | 1 | 2 | 3 | 4 | 5 |
| 32. I make efforts to change the current situation and move it in a positive direction. | 1 | 2 | 3 | 4 | 5 |
| 33. Before the final outcome is determined, I tend not to view the situation as overly serious. | 1 | 2 | 3 | 4 | 5 |
| 34. I have a more positive attitude toward my major-related coursework. | 1 | 2 | 3 | 4 | 5 |
| 35. I am more focused when completing academic tasks. | 1 | 2 | 3 | 4 | 5 |
| 36. I am better able to plan and manage my study time effectively. | 1 | 2 | 3 | 4 | 5 |
| 1. I should make my life more diverse rather than immersing myself in the online world. | 1 | 2 | 3 | 4 | 5 |
| 38. I am better able to control the amount of time I spend on social media apps such as Douyin/TikTok and Weibo. | 1 | 2 | 3 | 4 | 5 |
| 39. Playing video games is less likely to interfere with my ability to do other things. | 1 | 2 | 3 | 4 | 5 |
| 40. My impulse to immediately pick up my phone to check messages has decreased. | 1 | 2 | 3 | 4 | 5 |
| 41. I am better able to make use of various resources available at my university. | 1 | 2 | 3 | 4 | 5 |
| 42. I am more willing to participate in different activities to enrich my leisure time. | 1 | 2 | 3 | 4 | 5 |
| 43. I am better able to maintain a balance between my academic work and daily life. | 1 | 2 | 3 | 4 | 5 |
| 44. I am better able to find my own goals and direction within campus life. | 1 | 2 | 3 | 4 | 5 |
| 45. I feel more at ease when interacting with others. | 1 | 2 | 3 | 4 | 5 |
| 46. I can adapt more flexibly to different interpersonal communication situations. | 1 | 2 | 3 | 4 | 5 |
| 47. I am able to communicate with others more patiently. | 1 | 2 | 3 | 4 | 5 |
| 48. I can handle conflicts with others in a more appropriate manner. | 1 | 2 | 3 | 4 | 5 |
| 49. I am less likely to overthink how others view me. | 1 | 2 | 3 | 4 | 5 |
| 50. I am able to obtain emotional help and support from others (e.g., family, friends, teachers). | 1 | 2 | 3 | 4 | 5 |
| 51. I can perceive more friendliness and care from others. | 1 | 2 | 3 | 4 | 5 |
| 52. I am more willing to initiate communication with others. | 1 | 2 | 3 | 4 | 5 |
| 53. I am more willing to proactively contact new acquaintances or familiar people. | 1 | 2 | 3 | 4 | 5 |
| 54. When others ask me for help, I am more willing to put aside what I am doing to assist them. | 1 | 2 | 3 | 4 | 5 |
| 55. Even when I do not gain personal benefits, I am willing to lend a helping hand to others. | 1 | 2 | 3 | 4 | 5 |

**●**Subscale I: Emotional Feelings (12 items)

Psychological Positive Motivation: Items 1–3

Psychological Calmness: Items 4–6

Emotional Disturbance: Items 7–12

**●**Subscale II: Self-Cognition (10 items)

Competence Efficacy: Items 13–16

Self-Acceptance: Items 17–19

Body Self-Perception: Items 20–22

**●**Subscale III: Frustration Coping (11 items)

Concentration of Energy: Items 23–26

Flexible Thinking: Items 27–29

Optimistic Mindset: Items 30–33

**●**Subscale IV: Self-Control and Social Adaptation Efficacy (11 items)

Academic Coping: Items 34–36

Addiction Control: Items 37–40

Campus Adaptation: Items 41–44

**●**Subscale V: Interpersonal Interaction (11 items)

Interpersonal Communication: Items 45–48

Relationship Perception: Items 49–51

Willingness to Interact: Items 52–55
